# Non-invasive detection and monitoring of oral squamous cell carcinoma and oral potentially malignant disorders: an umbrella review on diagnostic accuracy

**DOI:** 10.1007/s00784-026-07109-x

**Published:** 2026-08-28

**Authors:** M. C. C. Groenevelt, L. Meulenbroeks, R. H. Brakenhoff, J. B. Poell, M. P. Fransen, A. H. Zwinderman, E. R. Brouns, L. W. P. Peute

**Affiliations:** 1 Amsterdam UMC, University of Amsterdam, department of Public and Occupational Health, Meibergdreef 9, Amsterdam, The Netherlands; 2 Amsterdam UMC, Vrije Universiteit Amsterdam, department of Otolaryngology/ Head and Neck Surgery, De Boelelaan 1117, Amsterdam, The Netherlands; 3 Cancer Center Amsterdam, Imaging and Biomarkers, Amsterdam, The Netherlands; 4 Cancer Center Amsterdam, Cancer Biology and Immunology, Amsterdam, The Netherlands; 5 Amsterdam UMC, University of Amsterdam, department of Epidemiology and Data Science, Meibergdreef 9, Amsterdam, The Netherlands; 6 Amsterdam UMC, Vrije Universiteit Amsterdam, department of Oral and Maxillofacial Surgery and Oral Pathology, Amsterdam, The Netherlands; 7 Amsterdam UMC, University of Amsterdam, department of Medical Informatics, Amsterdam, The Netherlands; 8 Amsterdam Public Health Research Institute, Quality of Care, Digital Health, Amsterdam, The Netherlands

**Keywords:** Oral potentially malignant disorders, Oral squamous cell carcinoma, Oral cancer, Diagnostic accuracy, Non-invasive diagnostic tests, Early detection

## Abstract

**Objectives:**

Various non-invasive diagnostic techniques have been developed and assessed to improve early detection and monitoring of oral squamous cell carcinoma and oral potentially malignant disorders. However, a clear overview of the diagnostic performance and their ability to detect clinically relevant changes, is lacking. This study evaluated clinically tested, non-invasive techniques for early detection and monitoring of oral squamous cell carcinoma and potentially malignant disorders, focusing on diagnostic accuracy.

**Methods:**

After a structured search strategy and critical selection of all relevant systematic reviews, data was extracted from 57 reviews in total, of which 29 were included for qualitative analysis, 20 for meta-analysis and eight for both qualitative and meta-analysis.

**Results:**

Among nine techniques that are available in practice, optical coherence tomography, spectroscopy, and cytology with oral brushes demonstrated the highest effectiveness. Optical coherence tomography demonstrated the highest diagnostic accuracy, with a sensitivity and specificity estimated at 0.91 and a diagnostic odds ratio of 108.5. Furthermore, when combined with artificial intelligence, diagnostic accuracy is even further improved.

**Conclusions:**

Most studies evaluating diagnostic accuracy of non-invasive diagnostic tests for potentially malignant disorders and oral squamous cell carcinoma grouped these conditions together without stratifying by lesion type. Therefore, no firm conclusions can be made about the diagnostic accuracy in detecting oral squamous cell carcinoma or different types of potentially malignant disorders. More high quality, primary studies should be conducted to specifically assess the diagnostic accuracy of different diagnostic tests per lesion type.

**Clinical relevance:**

No clinical guidelines are currently available for management of oral potentially malignant disorders, and practical guidelines for clinical decision making in the field of oral squamous cell carcinoma and oral potentially malignant disorders will be helpful. The results of this umbrella review can inform clinical practice, guideline development, and future research priorities.

**Supplementary Information:**

The online version contains supplementary material available at https://doi.org/10.1007/s00784-026-07109-x.

## Background

In recent years, various non-invasive diagnostic techniques have been developed and assessed to improve early detection and monitoring of oral squamous cell carcinoma (OSCC) and oral potentially malignant disorders (OPMDs). However, a clear overview of the evidence of the effectiveness of different techniques is lacking. Existing reviews comparing adjunctive non-invasive techniques do often not consider recent advancements in cytology, biomarker analysis and innovations in artificial intelligence and deep learning, but instead only focus on a subset of the available diagnostic tests [1]. To improve the development and implementation of a new non-invasive diagnostic test, it is important to know the effectiveness and accuracy of the current techniques and to translate this evidence into the current clinical practice of early detection of OSCC and/or OPMD [2].

OSCC predominantly originates from the mucosal lining of the oral cavity, and is typically preceded by precancerous changes in the mucosal epithelium. Some of these potential precancerous mucosal changes are visible as lesions and are classified as OPMDs. Common OPMDs include oral leukoplakia (OL), erythroplakia, proliferative verrucous leukoplakia (PVL), oral lichen planus (OLP) and oral submucous fibrosis -(OSF), with OL being the most frequently encountered OPMD amongst different demographics [3]. The reported rate of malignant transformation for OL varies widely, ranging from 1.1% to 40.8%, with the estimated time to development of OSCC spanning between 1.8 and 5.1 years [4].

Currently, the standard procedure to detect abnormalities in the oral mucosa and thereby OPMDs is through conventional oral examination (COE). However, the accuracy of using only COE as diagnostic method for the early detection of OPMDs remains limited [5, 6]. Besides a distinct risk of development into OSCC, OPMDs have their own clinical features, including color, texture, and size variations. The clinical presentation can be similar among OPMDs and are therefore hard to diagnose precisely [7–9]. The difficulty of defining the potential malignancy of these lesions lies in the fact that the OPMDs may remain invisible in the clinical presentation of the lesion, until its malignant progression [3, 6, 7].

Until this day, the gold standard in the diagnostic process of OPMD is a biopsy with histopathological assessment to determine the presence and grade of oral epithelial dysplasia [10, 11]. The histopathology of OPMD is characterized by microscopically altered cells that often show dysplastic features and harbor cancer-associated genetic alterations [3, 7]. Presence of dysplasia indicates a risk of malignant transformation. However, the time to transformation is highly variable and many dysplastic lesions do not transform [12]. As biopsy is an invasive procedure, it is usually performed by a specialist. Not only is this procedure uncomfortable for patients, but it also comes with inherent limitations. The biopsy site is essential for accurate histopathological examination and therefore it is sometimes recommended to perform multiple biopsies, especially in large heterogeneous lesions. Variations in available biopsy techniques and procedures may also lead to differences in diagnostic outcomes. Another complexity is that the pathological assessment of oral epithelial dysplasia is subjective and open to interpretation, which leads to inter-observer variability [7, 13].

With the oral cavity being easily accessible for inspection and sampling, non-invasive methods are attractive. In non-invasive diagnostic procedures, a method of measurement is used, which does not physically break the skin, or enters the body deeply through an external orifice. Medical imaging techniques are also considered as non-invasive [14]. In this study, techniques that may be considered minimally invasive were also classified as non-invasive, as they do not involve entry into the body and are limited to superficial cell sampling. Various non-invasive techniques, such as vital staining and other visualization techniques with or without imaging devices, have been developed and evaluated to improve the process of early detection, diagnosis, and monitoring of OSCC and OPMDs. In the monitoring of OSCC and OPMDs the patients’ condition is closely watched and certain exams and tests can be done, for example with non-invasive techniques [15]. In various studies the effectiveness both in primary and secondary care has been evaluated, aiming to determine whether these non-invasive techniques could replace biopsy as gold standard [16].

In the present study, the primary aim is to evaluate clinically tested, non-invasive techniques for the early detection and monitoring of OSCC and OPMDs, including the most recent innovations such as cytology, molecular biomarker analysis based on oral brush and saliva samples, and the additional use of deep learning- and Artificial Intelligence (AI)-models. Given the lack of quantitative synthesis in current literature, a secondary aim of this study is to provide evidence-based recommendations regarding the clinical application of these, in some cases, innovative approaches. These recommendations are intended to guide healthcare practices and to provide further research on evidence needed on promising innovative techniques for the early detection and monitoring of OSCC and OPMD.

### Research question

What is the effectiveness and accuracy of clinically tested non-invasive diagnostic techniques used for early detection and monitoring of oral squamous cell carcinoma and oral potentially malignant disorders?

## Methods

### Protocol and guidelines

This umbrella review was conducted following the 2020 Preferred Reporting Items for Systematic Reviews and Meta-Analyses (PRISMA) guidelines [17]. The study protocol was developed a priori and registered with the International Prospective Register of Systematic Reviews (PROSPERO) under registration number CRD42024587637. The crucial steps in this review, screening, and selection for title and abstract (CG & LP), screening for full-text eligibility, assessment of methodological quality and data-extraction (CG & LM) were performed by two reviewers independently using predefined criteria and forms. Disagreements were solved by consensus or arbitration of a third reviewer (LP).

### Search strategy

A structured search strategy (supplementary file 1) was designed to retrieve all relevant studies that evaluated the efficacy of non-invasive techniques in the early detection of OSCC and OPMDs. Involvement of a medical librarian ensured a comprehensive and methodologically sound literature search. PubMed, PsycINFO, and MEDLINE were systematically searched. No limitations were set on publication date or language. Individual searches were performed in July 2024. Additionally, reference lists of included studies were searched to identify relevant references that could have been missed in the initial search. Removal of duplicates in the search, screening and selection of the included articles was done using Rayyan [18].

### Eligibility criteria

Eligibility was assessed by PICOS [19]. Eligibility criteria were as follows:

- Systematic reviews (SRs) on diagnostic accuracy, with or without meta-analyses
- Studies that assessed the effectiveness of the detection of OSCC or OPMDs with non-invasive diagnostic techniques, in patients with OPMDs or OSCC
- Studies that concerned non-invasive screening techniques
- Studies with diagnostic accuracy outcome measures: sensitivity and specificity

Clinical (primary) studies, case-reports, umbrella reviews, non-original articles (summary reviews, editorials, letters, and comments), and unavailable or inaccessible full-text articles were excluded. Studies that reviewed invasive diagnostic tests, diagnostic tools that have not been tested in clinical practice or only included in-vitro testing were also excluded.

### Assessment of methodological quality

Critical appraisal of all included SRs was conducted using the JBI critical appraisal instrument for Systematic Reviews and Research Synthesis [20]. Discrepancies were solved upon mutual consensus and results were discussed with a third review author (LP). Studies were scored based on the number of questions answered with “Yes”. To ensure the inclusion of studies with at least moderate to high methodological quality, those scoring three or lower were excluded.

### Data extraction

Data was extracted from all included reviews using a data collection form. Data extracted were the study characteristics, type, and number of (primary) studies included, critical appraisal tools used and heterogeneity analysis. Data representing the different types of diagnostic techniques, the included study populations and the applied reference standards were taken into account. Data related to invasive diagnostic tests, and in-vitro or ex-vivo testing were not extracted. For study outcomes the data representing diagnostic accuracy were extracted: diagnostic odds ratio (DOR), sensitivity scores, specificity scores.

### Data synthesis

Data was independently extracted by two reviewers (MG & LM), and one consistent overview with the primary data was used for the final analysis [21, 22]. A descriptive approach was used for studies that provided only qualitative evidence on the outcome measures, as well as techniques for which the reported quantitative data was insufficient to perform a meta-analysis. Data synthesis was conducted based on the outcomes reported in the SRs and re-analysis of the primary studies was not performed.

A positive correlation between sensitivity and specificity among the included studies, violated the model assumptions of a HSROC analysis [23]. Therefore, for the quantitative analysis, a bivariate random-effects meta-analysis was conducted. Log DORs were estimated using a joint model of logit sensitivity and specificity, with random effects, to account for between-study heterogeneity and the correlation between sensitivity and specificity. DOR is a measure to estimate the discriminative power of a diagnostic tests and depends fully on sensitivity and specificity values [24]. A DOR of one means that the test has little discriminatory ability. A DOR greater than one indicates that the test can distinguish between those with- and without OSCC or OPMD. The higher the DOR values, the better the discriminatory test performance [25].

Sensitivity and specificity were jointly estimated using a bivariate random-effects model, accounting for their correlation and for clustering within studies [26]. To compare test performance, a Bayesian pairwise multivariate regression was conducted to estimate differences in log-sensitivity, log-specificity, and log-DOR, incorporating clustering (e.g., multiple index tests per study). This analysis used a Markov chain Monte Carlo (MCMC) method to model the underlying mathematical posterior distributions, based on the probability distributions [27]. Statistical significance was set at 0.01 to account for multiplicity.

Outcomes are reported as sensitivity, specificity and DORs, based on log-values. Analyses were performed in R version 4.3.2. using mvmeta version 1.0.3 [28] and rjags version 4.17. Heterogeneity was assessed using Cochran’s Q-test and I^2^ statistics, with fixed-effects models applied with a non-significant test, and a random-effect model otherwise [29]. Subgroup analysis for different lesion types (OSCC, OPMDs) was attempted to determine differential accuracy of the techniques for each lesion type.

### Strength of evidence

To evaluate the risk of publication bias at review level, Deeks’ regression test was applied, and results were visualized using a funnel plot. As this study analyzes SRs, the test specifically assesses small-study effects across reviews. Given the expected variability between sensitivity and specificity across studies, a simultaneous interpretation of two tests for funnel plot asymmetries will likely be challenging. Therefore, the analysis was performed based on the DOR, which summarizes test accuracy in one number. The slope of the regression line reflects the systematic differences between larger and smaller reviews, indicating possible review-level bias or methodological heterogeneity [30, 31]. To assess the overlap of the primary studies within the included SRs, a Corrected Covered Area (CCA) calculation was conducted, where an overlap of 0% - 5% is considered as “slight overlap”, 6 - 10% is considered as “moderate overlap”, 11% - 15% is considered as “high overlap” and a CCA of > 15% is considered as “very high overlap” [32].

## Results

### Results of the literature search

**Fig. 1:**
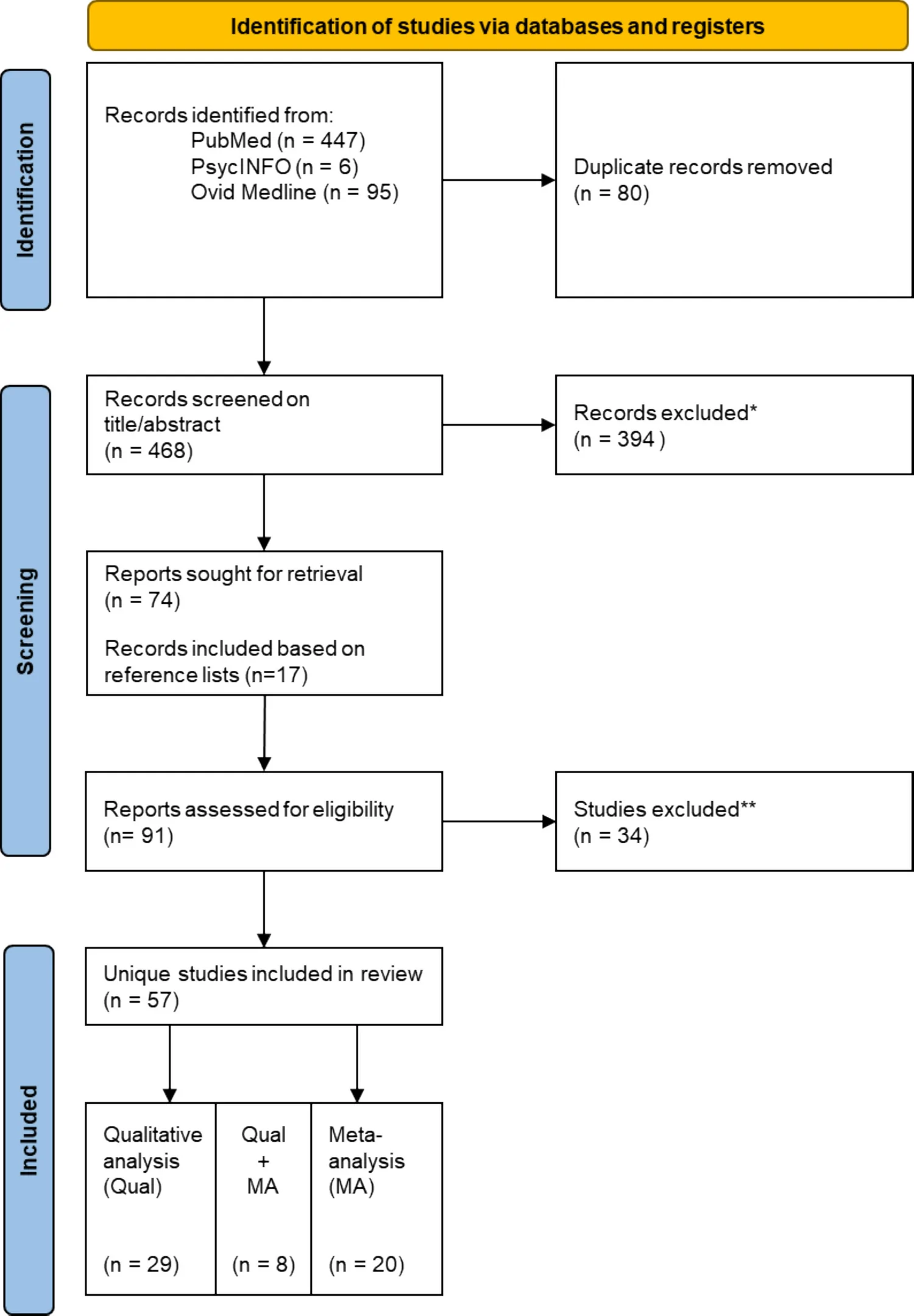
Flowchart of study selection. * Reasons for exclusion: incorrect study design (*n* = 9), unclear study design (*n* = 3), incorrect outcome (*n* = 193), incorrect population (*n* = 189). ** Reasons for exclusion: incorrect study design (*n* = 5), incorrect publication type (*n* = 10), incorrect outcome (*n* = 9), incorrect population (*n* = 1), discrepant results (*n* = 1), insufficient methodological quality (*n* = 9), unclear methodology (*n* = 1), incorrect inclusion (*n* = 1). *Qual * qualitative, *MA * meta-analysis

A total of 57 studies were eligible for inclusion, of which in total 37 were eligible for qualitative synthesis, and 28 studies for the quantitative analysis. Eight studies were eligible for both qualitative and quantitative data, which accounts for the discrepancy between the subtotals and the overall total. The results of the screening and selection are shown in the PRISMA flow diagram (Fig. 1) [33]. An overview of excluded studies (with reasons) is listed in supplementary file 2. Nine studies were excluded after critical assessment of methodological quality (supplementary file 3). Overlap of the primary studies is visualized in supplementary file 4. All non-invasive diagnostic techniques that were investigated in the included SRs are summarized in supplementary file 5, each accompanied by a brief description.

### Histopathological diagnostic criteria

Out of 57 included reviews, 16 evaluated only OSCC datasets, three only OPMD, and 38 studies both OSCC and OPMD. Most reviews defined their histopathological endpoints and those of the primary studies, but this was highly variable and sometimes these were not specified among the primary studies. Some studies classified all grades of dysplasia as histopathological positive results, while others restricted positivity only to high-grade (severe) dysplasia, carcinoma in situ, or squamous cell carcinoma. Some studies used other methods such as examination by a physician with specialist knowledge or training with long-term follow up as suitable reference standard for participants who screened negative [34–36]. This inconsistency limits the ability to assess the test performance in distinguishing lesions with high risk of malignant transformation from those with a lower risk [37].

### Qualitative analysis

In total 37 studies were included for qualitative analysis. The techniques were subdivided into six distinct groups based on their characteristics: oral examination, vital staining, chemiluminescence, autofluorescence, imaging-based methods, and cytology and molecular biomarkers. In addition, the use of AI adjunctive to these techniques was described. Conventional oral examination, toluidine blue, oral brush cytology, chemiluminescence, and autofluorescence were assessed in both primary and specialist care, whereas narrow-band imaging, confocal microscopy, optical imaging, and AI were mainly evaluated in specialist or unreported settings. Most reviews included patients with OPMDs and/or OSCC, with inconsistent reporting of healthy controls. Evidence from OPMD-only or OSCC-only populations was limited (Table 1). Study characteristics are shown in Table 1 and Table 2. Table 2 shows SRs that investigated biomarkers for the diagnosis of OPMD and/or OSCC.

**Table 1:** Study characteristics of systematic reviews included for qualitative analysis

Author (year)	Number of included studies [datasets]; Diagnostic setting	Diagnostic techniques	Sensitivity (single, pooled, or ranged values) [95% CI]	Specificity (single, pooled, or ranged values) [95% CI]	Diagnostic odds ratio (single, pooled, or ranged values) [95% CI]	Descriptive conclusions
*Study population: OPMD and OSCC*						
Patton [38]	36 Primary care, Specialist care	COE	0.71 to 0.95	0.64 to 0.99	NR	• Screening low risk populations for OSCC may have little benefit • TB is better in detecting OSCC than precancerous dysplastic lesions • Better education for examiners improves diagnostic accuracy
		TB	0.72 to 1.00	0.45 to 0.93	NR	
		Oral brush	1.00	0.93 and 1.00	NR	
		Computer-based techniques (adjunctive to other diagnostic techniques)	0.80	0.77	NR	
Patton [37]	23 Primary care, specialist care	COE	0.40 to 0.93	0.50 to 0.75	NR	• TB is effective as a diagnostic adjunct in assessing high-risk mucosal lesions • Oral brush (OralCDx) improves detection of dysplastic changes in clinically suspicious lesions
		TB	0.38 to 0.98	0.09 to 0.93	NR	
		Oral brush	0.71 to 1.00	0.27 to 0.94	NR	
		CHEM (ViziLite; ViziLite plus)	1.00; NR	0 to 0.14; NR	NR	
		AF (VELscope)	0.98 and 1.00	0.78 and 1.00	NR	
Walsh [34]	13 Primary care, Specialist care	COE	0.50 [0.07–0.93] to 0.99 [0.97–1.00] †; 0.95 [0.92–0.97] ‡; 0.97 [0.96–0.98] ⁰	0.98 [0.97–1.00] to 0.99 [0.99–0.99]†; 0.81 [0.79–0.83] ‡; 0.75 [0.73–0.77] ⁰	NR	• Mouth self-examination is less sensitive than COE • Diagnostic techniques were better at classifying absence of OPMD or OSCC in disease-free individuals than classifying presence in diseased individuals
		COE vs COE + TB	0.50 [0.12–0.88] vs 0.40 [0.05–0.85]	0.92 [0.91–0.93] vs 0.91 [0.90–0.91]	NR	
		Mouth self-examination	0.18 [0.13–0.24] and 0.33 [0.10–0.65]	1.00 [1.00 −1.00] and 0.54 [0.37–0.69]	NR	
Omar [39]	121 Specialist care	Oral brush	0.71 to > 0.90 ^	0.32 to > 0.90 ^	NR	• Due to subjectivity and dependence on examiner experience, TB appears to be unreliable • Oral brush (OralCDx) is at least as sensitive as a tissue biopsy in detecting dysplasia or cancer
		TB	0.94 to 0.98	0.73 to 0.93	NR	
		AF (VELscope)	0.97	0.94	NR	
		OCT	0.93	0.93	NR	
		ESS	0.64 and 0.96	0.90 and 0.96	NR	
		RS	0.76 to 1.00 ^	0.77 to > 0.90 ^	NR	
		AF spectroscopy	0.83 to 0.90	0.79 to 0.89	NR	
Rashid [40]	25 Specialist care, Primary care	CHEM (ViziLite; ViziLite Plus; Microlux DL)	0.77 to 1.00; 0 to 0.59; 0.78	0.00 to 0.28; 0.76 to 0.78; 0.71	NR	• Inadequate evidence to confirm the effectiveness of chemiluminescence and autofluorescence imaging devices as screening adjuncts • CL and AF are more suited for specialist clinics with higher disease prevalence and experienced clinicians
		AF (VELscope)	0.30 to 1.00	0.15 to 1.00	NR	
Ye [41]	5 NR	DNA-ICM	0.89 [0.83–0.94]	0.99 [0.97–1.00]	446.08 [73.36–2712.43]	• In diagnosis of OPMD and OSCC, DNA-ICM is more accurate than OralCDx brush
Lingen [42]	8 Specialist care	AF	0.50 [0.21–0.79]	0.39 [0.31–0.47]	NR	• Oral brush most accurate adjunct, but high false-positive rate and significant concerns about bias and evidence reliability • Clinicians should be cautious about the potential benefit of any adjunct in clinical practice
		CHEM	0.00 [0.00–0.60]	0.76 [0.66–0.84]	NR	
		CHEM + TB (ViziLite Plus)	0.81 [0.71–0.89]	0.69 [0.63–0.75]	NR	
		Oral brush	0.96 [0.81–1.00]	0.90 [0.79–0.97]	NR	
		Oral brush + TB	0.95 [0.86–0.99]	0.68 [0.56–0.78]	NR	
Alsarraf [43]	36 NR	Oral brush (OralCDx1; cytobrush; toothbrush)	0.71 to 1.00 ^; 0.69 to 1.00 (with liquid to based cytology: 0.87 to 0.98) ^; 0.77 to 0.83 (with liquid to based cytology: 0.75) ^	0.24 to 1.00 ^; 0.52 to 1.00 (with liquid to based cytology: 0.69 to 0.95) ^; 0.50 to 1.00 (with liquid to based cytology: 0.50)^	NR	• Oral brush with liquid-based technology offers significant advantages compared to conventional cytology
Ansari [36]	3 NR	NBI	0.89 to 0.99 ^	0.85 to 1.00 ^	NR	• In detecting severe dysplasia and OSCC, NBI is a promising diagnostic tool • It outperforms oral examination and white light endoscopy in diagnostic accuracy • NBI requires specialist training
Tiwari [44]	27 Primary care, Specialist care	COE	0.17 to 0.99*; 0.06 to 0.97**	0.33 to 0.99*; 0.43 to 0.98**	NR	• Adjunctive AF showed varying effectiveness in detecting OPMD/OSCC • Adjunctive optical fluorescence imaging by AF can improve visualization of oral mucosal lesions
		AF	0.30 to 1.00**	0.13 to 0.93**	NR	
		COE + AF	0.74 to 1.00*; 0.46 to 1.00**	0.38 to 0.98*; 0.06 to 0.74**	NR	
Mazur [45]	43 NR	AF	0.00 to 1.00^	0.13 to 1.00^	NR	• Due to variation in data across studies, obtaining reliable comparisons is difficult • The best performance was seen with NBI and vital staining
		HRME	0.73 to 0.95^	0.86 to 1.00^	NR	
		Optical spectroscopy	0.53 to 0.99^	0.63 to 0.96^	NR	
		NBI	0.85 to 0.94^	0.80 to 0.95^	NR	
		Vital staining	0.83 to 1.00^	0.43 to 0.84^	NR	
Saraniti [46]	9 Primary care, specialist care	WLE	0.41 to 0.96^	0.60 to 0.79^	NR	• NBI is an effective technique for the early detection of OSCC and OPMD • Adequate training is essential for NBI
		NBI	15.39 to 99.00^	55.01 to 100^	NR	
Walsh [47]	NR [5] Specialist care	DNA-ICM	0.76 [0.68–0.82]	0.98 [0.72–0.99]	NR	• Oral cytology was the most potential technique • Combined adjunctive tests involving cytology require further investigation
Walsh [35]	18 Specialist care	COE	0.50 [0.07–0.93] to 0.99 [0.97–1.00]•; 0.94 [0.90–0.97] to 0.97 [0.96–0.98]••	0.94 [0.88- 0.97] to 0.99 [0.98–1.00]•; 0.75 [0.73–0.77] to 0.98 [0.98–0.99]••	NR	• COE for screening for OPMD and OSCC shows variable sensitivity, and a consistently high specificity
		COE vs COE + TB	0.50 [0.12–0.88] vs 0.40 [0.05–0.85]	0.92 [0.91–0.93] vs 0.91 [0.90–0.91]	NR	
		Mouth self-examination	0.09 to 0.43 ^	0.44 to 1.00 ^	NR	
		Mobile applications for remote screening	0.82 to 0.94 ^	0.72 to 1.00 ^	NR	
Essat [48]	4 Specialist care	COE	0.40 to 0.82^	0.32 to 0.68^	NR	• The highest sensitivity and specificity were found in COE conducted by hospital-based dentists, followed by primary care dentists, hygienists/therapists, and dental nurses
Mendonca [49]	3 NR	AF (5-ALA induced protoporphyrin IX fluorescence)	0.91 [0.82–0.96]	0.78 [0.71–0.85]	44.61 [7.11–280.07]	• Superior accuracy levels in the diagnosis of OSCC compared to OPMDs • Not enough evidence to consider these AF techniques for definitive diagnosis of OPMD or OSCC
Ramani [50]	1 NR	CLE—qualitative assessment	1	0.93	NR	• Reflectance confocal microscopy reveals characteristic features of OSCC, including cellular and architectural disarray, keratin pearls, cellular pleomorphism, and increased nuclear cytoplasmic ratios • Diagnostic utility is limited by poor depth penetration, strong backscatter of light in highly keratinized tissue, and signal diffusion from dense inflammation
Kim [51]	12 NR	AI (adjunctive to other diagnostic techniques)	0.91 [0.87–0.94]	0.88 [0.84–0.92]	63.02 [40.32–98.49]	• Diagnostic power of AI-assisted screening of precancerous and cancerous lesions would be overestimated • Clinicians should be careful when interpreting these outcomes
Beristain-Colorado [52]	9 NR	CCN + CLE	0.87	0.90	NR	• AI can aid early diagnosis, especially for clinicians in remote locations with limited access to specialist oral pathology advice • Convolutional neural networks (CNNs) in combination with photographic techniques show promise for early OSCC detection
		CNN + HRME	0.75	0.89	NR	
		CNN + HSI	0.94	0.98	NR	
		CNN + OCT	0.93	0.74	NR	
		CNN + photography	0.87 to 0.99 ^	0.85 to 1.00 ^	NR	
Li [53]	NR [16]	AI (adjunctive to other diagnostic techniques)	0.90 [0.87–0.92]	0.89 [0.85–0.92]	68.44 [39.48–118.62]	• High diagnostic sensitivity, suggesting a satisfactory level of diagnostic accuracy in screening for OPMD and oral mucosal cancerous lesions
	7	AI + AF	NR	NR	27.09 [17.85–41.11]	• AF shows the lowest accuracy compared to other techniques such as photography
	NR [7]	AI + OCT	NR	NR	154.06 [38.83–611.34]	• OCT is found to be a satisfactory diagnostic image tool
	10 NR	AI + photography	NR	NR	61.83 [33.42–114.39]	• No significant differences were found • Potential superior performance of clinical photography in auto-detecting OPMD and oral cancerous lesions are highlighted
*Study population: OPMD*						
Datta [54]	11 Specialist care	DNA-ICM	0.16 to 0.96	0.90 to 1.00	NR	• Limited evidence for the use of DNA-ICM as an adjunct OSCC screening tool
Mendonca [49]	3 NR	AF (fluorescence spectroscopy)	0.74 [0.60–0.85]	0.96 [0.91–0.98]	57.84 [19.52–171.30]	• Superior accuracy levels in the diagnosis of OSCC as compared to OPMDs • Not enough evidence to consider these AF techniques for definitive diagnosis of OPMD or OSCC
Ramani [50]	4 NR	CLE—qualitative assessment	0.50 to 0.80 ^	0.79 to 1.00 ^	NR	• Confocal microscopy images in the diagnosis of OPMD showed a higher diagnostic accuracy when conducted by more experienced examiners
*Study population: OSCC*						
Alabi [55]	41 NR	CNN (adjunctive to other diagnostic techniques)	0.75 to 0.99	0.81 to 0.95	NR	• Machine learning techniques for diagnosing and predicting OSCC outcomes offer clinicians a more precise and data-driven approach to decision-making • Significant challenges remain in integrating these methods into routine clinical practice
		ANN (adjunctive to other diagnostic techniques)	0.71 to 0.80	0.77 to 0.98	NR	
		SVM (adjunctive to other diagnostic techniques)	0.91	0.96	NR	
Lima [56]	45 NR	AF (VELscope, Identafi, Evince)	0.33 to 1.00; 0.50; 1.00	0.12 to 0.89; 0.81; 0.91	NR	• AF and fluorescent probes can aid dentists to accurately diagnose OSCC during daily clinical activity, but it cannot yet replace histopathological examination
		CHEM (ViziLite)	0.00	0.75	NR	
		Fluorescent spectroscopy/probes (5-ALA, hypericin, rhodamin 610, porphyrin)	0.90 to 1.00; 1.00; 0.88; 1.00	0.51 to 0.96; 0.75 to 1.00; 1.00; 1.00	NR	
Sethi [57]	3 NR	CLE	0.83 to 0.99	0.80 to 1.00	NR	• CLE may aid in the diagnosis of OSCC, but more studies with consistent methods are required to enable reliable comparisons
Kim [51]	7 NR	AI + AF	0.90 [0.83–0.94]	0.82 [0.44–0.96]	25.91 [6.31–106.45]	• AI analysis of images in cancer diagnosis is thought to be helpful in making fast decisions regarding further examination and treatment • Discriminating between precancerous lesions and normal tissues showed a high sensitivity of over 90%, showing good accuracy as a screening method
		AI + photography	0.91 [0.75–0.98]	1.00 [0.29–1.00]	431.65 [4.00–46537.47]	
		AI + OCT	0.94 [0.85–0.98]	0.95 [0.79–0.99]	2612.00 [14.71–4666.35]	
Mendonca [49]	16 NR	AF (VELscope ao)	0.96 [0.94–0.98]	0.58 [0.55–0.61]	NR	• Superior accuracy levels in the diagnosis of OSCC as compared to OPMDs • Not enough evidence to consider these AF techniques for definitive diagnosis of OPMD or OSCC
		AF (fluorescence spectroscopy)	0.93 [0.88–0.97]	0.97 [0.94–0.99]	NR	
Ramani [50]	6 NR	CLE—qualitative assessment	0.86 to 0.95 ^	0.78 to 1.00 ^	NR	• Quantitative approaches with machine-assisted precision show promise for future use of CLE
		CLE—quantitative assessment #	0.72	0.85	NR	
Elmakaty [58]	27 NR	AI (adjunctive to other diagnostic techniques)	0.92 [0.87–0.95]	0.92 [0.87–0.95]	132.00 [62.60–277.00]	• High accuracy in differentiating healthy oral tissue from OSCC

*OPMD* oral potentially malignant disorders, *OSCC *oral squamous cell carcinoma, *95%CI *95% confidence interval, *NR *not reported, *COE *conventional oral examination, *TB *toluidine blue staining, *CHEM* chemiluminescence, *AF *autofluorescence, *OCT *optical coherence tomography, *ESS* elastic scattering spectroscopy, *RS *Raman spectroscopy, *DNA-ICM * DNA image cytometry, *NBI *narrow-band imaging, *HRME *high resolution micro endoscopy, *WLE *white light endoscopy, *CLE* confocal laser endomicroscopy, *HSI *hyperspectral imaging, *CNN *convolutional neural networks, *ANN *Artificial Neural Networks, *SVM *Support Vector Machines, *AI *artificial intelligence

Symbols:

† Studies with prevalence ≤ 10%

‡ Study with prevalence of 22%

⁰ study with prevalence of 51%

* Detecting OPMD or OSCC

** discriminating between benign, dysplastic, and neoplastic oral lesions

• studies with low prevalence

•• studies with high prevalence

# Epithelial cell sizes by automated cell order segmentation and distance map between cell borders

^ Range was manually calculated based on available data in the article

## Estimates were rounded to two decimal places

**Table 2:** Overview of biomarkers used in systematic reviews

Author (year)	Study population; diagnostic setting	Number of included studies	Methylation markers	mRNA	miRNA	Proteins	Metabolites	Descriptive conclusions
Guerra [59]	OSCC NR	12					x	• Combining salivary biomarkers improves diagnostic accuracy compared to individual markers • Salivary biomarkers were better at detecting early stage OSCC
Omar [39]	OPMD, OSCC Specialist care	42		x		x		• Salivary-based analysis is an accurate method with high sensitivity and reliable specificity for OSCC diagnosis • Easy to perform in primary care as it does not require special experience for interpretation of the information obtained
Tian [60]	OSCC NR	10			x			• miRNAs showed higher sensitivity and specificity in saliva compared to blood/serum samples • Their moderate accuracy suggests that miRNAs can be used to confirm or to exclude suspected cases
Maheswari [61]	OPMD, OSCC NR	6			x			• miRNA-27b showed the highest diagnostic value for OSCC, with studies supporting the potential of salivary miRNAs for early detection of malignancy in OPMD • Several miRNAs were upregulated or downregulated in OSCC and OPMD compared to healthy controls, highlighting their promise as non-invasive biomarkers
Rapado-González [62]	OSCC NR	16			x			• Salivary miRNAs showed high diagnostic accuracy
AlAli [63]	OSCC Specialist setting	6				x		• Because of limited strong, high-quality evidence, there is still uncertainty about the use of individual salivary biomarkers for the detection of OSCC
Assad [64]	OSCC NR	18					x	• Combination of choline, betaine, pipecolinic acid, and L-carnitine showed the highest sensitivity and specificity for OSCC diagnosis • As a single metabolite, choline, pipecolinic acid, propionyl choline, and S-sarboxymethyl-L-cysteine effectively distinguished early OSCC from healthy controls
Adeoye [65]	OPMD, OSCC NR	22	x*					• DNA hypermethylation markers, especially when combined, evaluated in saliva and oral swabs are more specific than sensitive for diagnosis of OSCC
Kang [66]	OSCC NR	11			x			• Salivary miRNA, detected via RT-qPCR, could be a promising non-invasive biomarker for OSCC diagnosis
Liu [67]	OSCC NR	20			x			• This study shows a moderate diagnostic accuracy of salivary miRNAs for OSCC
Rapado-González [68]	OSCC NR	10	x					• Saliva-based methylation markers show high diagnostic specificity, making it useful for diagnosis • New techniques like next-generation sequencing and digital PCR may improve sensitivity of these assays
Shaw [69]	OSCC NR	18		x	x	x		• Highest sensitivity and specificity were observed for mRNA and miRNA measured by PCR • Lowest sensitivity and specificity were found for IL-1B measured by ELISA • This study supports the potential of salivary biomarkers analyzed by PCR and/or ELISA for early cancer screening and diagnosis
Gaba [70]	OSCC NR	17		x	x	x		• Highest sensitivity and specificity for early detection of OSCC for DUSP-1 and S100P mRNA • Depends on availability of expertly trained clinicians able to run and interpret the analyses

*Saliva- and oral swab-based, *PCR* polymerase chain reaction, *RT-qPCR* real-time quantitative PCR, *ELISA* enzyme-linked immunosorbent assay, *NR* not reported

### Oral examination

COE was investigated in seven studies [34, 35, 37, 38, 44, 48, 71]. Studies show variable sensitivity and specificity. Both OSCC and OPMDs were analyzed, although different cut-offs for defining the lesions were used. Essat et al. found that COE for OSCC and OPMD conducted by hospital-based dentists delivers the highest sensitivity and specificity (0.90 and 0.76 resp.), which is followed by primary care dentists (0.81 and 0.73 resp.), hygienists/therapists (0.77 and 0.69 resp.) and dental nurses (0.68 and 0.59 resp.) [48]. Walsh et al. also investigated self-examination of the mouth, which showed a lower sensitivity compared to COE, for both OSCC and OPMD [34, 35].

### Vital staining

Vital staining can be performed with toluidine blue (TB), Rose Bengal, or Lugol’s iodine and was investigated in seven studies, solely or in addition to other techniques such as COE or oral brush cytology [34, 35, 37–39, 42, 45]. Mazut et al. reviewed several different techniques (autofluorescence (AF), high resolution micro endoscopy (HRME), narrow band imaging (NBI), optical spectroscopy, vital staining (VS)) and concluded that vital staining, together with NBI, showed the best performance [45]. Most studies used TB staining and Patton et al. stated in 2003 that this method is more effective for the detection of OSCC than for precancerous dysplastic lesions [38]. However, in 2008, they stated that TB is also effective in assessing high-risk mucosal lesions [37]. TB can be used as a diagnostic adjunct, but due to subjectivity and dependence on examiner experience, Omar et al. concluded that TB is an unreliable technique for OSCC and OPMD [39]. Walsh et al. compared the diagnostic accuracy of COE alone or with TB staining for the detection of OSCC and OPMD, and showed that this combination did not improve the sensitivity [35]. They also emphasized better education for examiners to improve diagnostic accuracy.

### Light-based methods: chemiluminescence

Four studies analyzed the use of chemiluminescence techniques for the diagnosis of OPMD and/or OSCC [37, 40, 42, 56]. Methods that are used for chemiluminescence imaging include ViziLite, ViziLite Plus and Microlux DL. ViziLite can be combined with TB staining, which is commercially available as a kit called ViziLite Plus. ViziLite is no longer available as standalone technique. Rashid et al. investigated the use of all three methods and they stated that there is not enough evidence to confirm the effectiveness of chemiluminescence imaging devices as screening adjuncts [40]. Their suggestion was that these tools are better suited to specialist clinics with higher disease prevalence and experienced clinicians. In addition, chemiluminescence preferentially detects keratotic lesions, rather than red lesions such as erythroplakias, and it also detects benign lesions resulting in more false positive results when distinguishing between OPMDs and benign tissues. The use of chemiluminescence additional to COE for early detection of OPMD or OSCC is not convincing [40].

### Light-based methods: autofluorescence

Autofluorescence (AF) methods were evaluated in 8 SRs [37, 39, 40, 42, 44, 45, 49, 56]. Among the studies included, VELscope was the most used commercial product, while other products, such as Identafi or Evince were only investigated in one or a few SRs. Other AF methods use fluorescent spectroscopy probes, such as 5-ALA, hypericin, rhodamin 610, and porphyrin. Studies highlighted the potential of AF and fluorescent probes to aid in the diagnosis of OPMDs and OSCC in addition to COE, but it cannot yet replace histopathological examination [44, 45, 56]. In contrast, other SRs concluded that there is not enough evidence to consider AF imaging techniques as screening adjuncts for diagnosis of OPMD or OSCC [40, 49]. Mazur et al. highlighted the importance of skilled examiners to prevent misinterpretation of the AF test and therefore, high false-positive results [45]. Two SRs investigated the use of AI adjunctive to AF and both showed that AI-assisted AF has a lower diagnostic accuracy compared to AI-assisted photography or OCT [51, 53].

### Imaging-based methods

Many different imaging techniques for the detection of OPMD and OSCC were discussed in the included reviews. Optical imaging techniques include white light endoscopy (WLE), optical coherence tomography (OCT), confocal laser endomicroscopy (CLE), HRME, NBI, magnetic resonance imaging (MRI), and spectroscopy (elastic scattering spectroscopy (ESS), Raman spectroscopy (RS)). Most of these techniques are only reported in one SR. NBI is studied in 3 SRs [36, 45, 46] and CLE in 2 SRs [50, 57]. NBI is a promising tool for the detection of OPMD (mainly severe dysplasia) and OSCC but requires adequate training of the specialists [36, 46]. Ramani et al. used a qualitative and a quantitative approach, with machine-assisted precision, for CLE [50]. Diagnostic utility was limited due to poor depth penetration, strong backscatter of light in highly keratinized tissue, and signal diffusion from dense inflammation. The diagnostic accuracy was higher when conducted by more experienced examiners. However, they stated that the quantitative approach shows promise for future use of CLE.

Several studies concluded that the use of mobile applications or artificial intelligence (AI) with photography can aid in early diagnosis, especially for clinicians in remote areas with limited access to specialist oral pathology advice [35, 51–53]. One SR emphasized that machine learning enhances OSCC diagnosis and prognosis by offering more precise, data-driven clinical decisions. However, clinical implementation remains challenging and they noted that AI diagnosis using OCT images has sufficient added value as a screening method for OPMDs [55]. Another SR showed in a subgroup analysis that AI-assisted OCT has a higher diagnostic accuracy and negative predictive value compared to AI-assisted photographic images or AF [51].

### Cytology and molecular biomarkers

Five reviews evaluated cytology using an oral brush [37–39, 42, 43]. Oral brush with liquid-based technology showed significant advantages compared to conventional cytology and this SR recommended a clear protocol for cytological sampling methods and clear criteria for diagnosis [43]. Another SR concluded that the oral brush is the most accurate adjunct, but with a high false-positive rate [42]. The reliability of the evidence in those studies was questioned. Two other studies showed that this method detects dysplasia in clinically suspicious lesions with a sensitivity comparable to tissue biopsy [37, 39]. Molecular biomarkers were based on saliva or oral brush samples, and DNA-image cytometry (DNA-ICM) based on oral brush samples. DNA-ICM showed higher accuracy than the OralCDx brush for diagnosis of OPMD and OSCC but with limited evidence for the adjunctive use in OSCC detection [41, 47, 54]. The different biomarkers analyzed in the reviews are divided into five groups (Table  2). DNA hypermethylation markers showed higher specificity compared to sensitivity in both saliva and oral swabs for diagnosis of OSCC, especially with a combination of multiple methylation markers. This combination also facilitated better discrimination of OSCC from OPMD or benign cases [65]. Rapado-González showed that saliva-based methylation markers have high specificity values for OSCC, making it promising for diagnosis. Innovative techniques, such as next-generation sequencing and digital PCR, may improve sensitivity of these biomarker assays [68]. Maheswari et al. concluded that miRNAs are promising markers for detecting early malignancy in OPMD and several miRNAs were found upregulated or downregulated in OSCC and OPMD compared to healthy controls [47]. Two SRs emphasized that a combination of multiple metabolite biomarkers improves the diagnostic accuracy compared to individual markers [59, 64].

### Quantitative analysis

A total of 28 reviews were included for quantitative analysis. Study characteristics are shown in Table 3. For the meta-analysis, techniques were subdivided into nine distinct groups based on their characteristics: conventional oral examination, vital staining, chemiluminescence, autofluorescence, optical imaging, imaging-based techniques, spectroscopy-based techniques, cytology-brush techniques, and computer-based techniques.

**Table 3:** Study characteristics of systematic reviews included for quantitative analysis

Author (year)	Study population;diagnostic setting	No. of included studies [datasets]	Diagnostic techniques		No. of patients	No. of lesions		Sensitivity (95% CI)		Specificity (95% CI)	DOR	Descriptive conclusions
*Conventional Oral Examination*												
Epstein [5]	OPMD, OSCC NR	24	COE		7079	1956	0.93 (0.91–0.94)		0.31 (0.28–0.34)		6.10 (2.10–17.60)	• Sensitivity was good (0.93) • Specificity was relatively poor (0.31) • It seems unlikely to rule out dysplasia or OSCC in asymptomatic patients who did not have recognized risk factors for cancer
Fuller [72]	OPMD, OSCC NR	3	COE		NR	149	0.89 (0.79–0.95)		0.61 (0.12–0.98)		NR	• COE has a significantly better accuracy compared to TB
Buenahora [73]	OPMD, OSCC NR	16	COE		2835	1892	0.63 (0.45–0.78)		0.78 (0.65–0.87)		6.00 (3.00–14.00)	• Highest specificity compared to chemiluminescence and autofluorescence for identifying lesions without dysplasia or changes in the malignancy of oral epithelium
Essat [48]	OPMD Specialist care	14	COE		NR	4668	0.71 (0.57–0.81)		0.85 (0.68–0.94)		NR	• Depending on the healthcare worker performing the assessment, sensitivity and specificity varies
Kim [74]	OPMD, OSCC NR	11	COE		705	1097	0.76 (0.63–0.86)		0.80 (0.59–0.92)		NR	• No significant differences between COE, cytology, and TB staining
Sulaiman [75]	OPMD, OSCC Primary care	5	COE		10069	NR	0.89 (0.72–0.96)		0.92 (0.78–0.97)		NR	• High level of pooled sensitivity and specificity • High level of accuracy in detecting precancerous and cancerous oral lesions by FHW
Gurushanth [71]	OPMD, OSCC Primary care	6	COE		110171	80505	0.75 (0.74–0.76)		0.97 (0.97–0.97)		131.84 (18.50–939.48)	• Visual screening is effective in identifying the target condition OPMDs or OSCC
*Vital staining*												
Fuller [72]	OPMD, OSCC NR	NR [8]	TB	NR		589	0.82 (0.64–0.95)		0.53 (0.37–0.69)		NR	• No improvement of oral examination by a cancer specialist
Macey [76]	OPMD, OSCC Specialist care	14	TB, LI, RB		1248	1338	0.84 (0.74–0.90)		0.70 (0.59–0.79)		NR	• Should be confined for use by expert clinicians • Should be used to facilitate biopsy site selection in heterogeneous PMD’s or as a surveillance tool
Lingen [42]	OPMD, OSCC Specialist care	15	TB		NR	1453	0.87 (0.80–0.94)		0.71 (0.61–0.82)		NR	• When used in primary care settings, more false-positive than true-positive results are shown
Kim [77]	OPMD, OSCC NR	27	TB		1921	2020	0.73 (0.65–0.79)		0.70 (0.63–0.75)		7.02 (4.54–10.84)	• No difference between COE and TB in patients with obvious neoplastic lesions • In identifying suspicious lesions, TB is more sensitive
Walsh [47]	OPMD, OSCC Specialist care	NR [14]	TB		NR	1780	0.86 (0.79–0.90)		0.68 (0.58–0.77)		NR	• Estimated values are based on low to very low certainty of evidence • Vital staining cannot be recommended as a replacement for histological assessment
Kim [74]	OPMD, OSCC NR	14	TB		705	695	0.71 (0.61–0.80)		0.81 (0.68–0.90)		NR	• TB has a higher diagnostic accuracy than clinical examination, but differences are not significant • TB should not be a replacement for conventional screening tests
*Light-based – Chemiluminescence*												
Lingen [42]	OPMD, OSCC Specialist care	5	ViziLite, ViziLite Plus, Microlux DL		NR	390	0.72 (0.62–0.81)		0.31 (0.25–0.36)		NR	• When used in primary care settings, the use of chemiluminescence would result in more false-positive diagnosis
Buenahora [73]	OPMD, OSCC NR	15	ViziLite		2399	1353	0.67 (0.38–0.87)		0.48 (0.28–0.69)		2.00 (1.00–5.00)	• A low capacity for diagnostic discrimination is shown
Moffa [78]	OPMD, OSCC NR	9	ViziLite, Microlux DL		582	555	0.85 (0.67–0.97)		0.52 (0.37–0.66)		8.59 (2.11–22.38)	• Chemiluminescence seems to be an adequate adjunctive tool, even if the lesion is not suspected at COE • The use of chemiluminescence improves the detection of early-stage tumors
Walsh [47]	OPMD, OSCC Specialist care	NR [6]	CHEM		NR	432	0.94 (0.35–0.99)		0.19 (0.03–0.67)		NR	• Should be confined for use by expert clinicians • Should be used to facilitate biopsy site selection in heterogeneous OPMDs or as a surveillance tool
Kim [79]	OPMD, OSCC NR	16	CHEM		NR	NR	0.85 (0.69–0.92)		0.43 (0.22–0.67)		4.06 (1.53–10.80)	• There would be no difference between chemiluminescence and clinical examination in evaluating obvious neoplastic lesions
Shaw [80]	OPMD (OL) NR	11	CHEM		906	NR	0.75 (0.29–0.98)		0.98 (0.91–1.00)		9.63 (1.08–85.98)	• Chemiluminescence can be used as an alternative diagnostic adjunct to biopsy for early screening and diagnosis of various OPMDs • It can be useful as secondary level of prevention for early OSCC
	OPMD (OLP) NR	7	CHEM		439	NR	0.78 (0.18–1.00)		0.60 (0.11–0.96)		3.02 (0.21–44.38)	
	OPMD (OSF) NR	5	CHEM		438	NR	0.89 (0.13–1.00)		0.76 (0.12–1.00)		4.12 (0.22–77.63)	
Kim [74]	OPMD, OSCC NR	11	CHEM		731	768	0.88 (0.76–0.94)		0.57 (0.29–0.81)		NR	• Chemiluminescence showed similar adjunctive diagnostic power as autofluorescence • Chemiluminescence showed the highest sensitivity compared with visual examination
Mendonca [49]	OPMD, OSCC NR	7	ViziLite		550	495	0.74 (0.68–0.79)		0.47 (0.40–0.53)		5.10 (0.90–28.94)	• In identifying dysplasia, average sensitivity and low specificity was obtained
*Light-based – Autofluorescence*												
Fuller [72]	OPMD, OSCC NR	NR [4]	AF, LIAF		NR	140	0.98 (0.93–1.00)		0.84 (0.65–0.95)		NR	• Sensitivity and overall accuracy were impressive and outperformed COE, TB and cytology • Generalizability of this data is limited
Luo [81]	OPMD, OSCC Specialist care	16	AF		NR	1693	0.88 (0.77–0.94)		0.62 (0.41–0.79)		11.52 (3.10–42.87)	• The use of autofluorescence is more proper for specialists
Lingen [42]	OPMD, OSCC Specialist care	7	VELscope, OralID		NR	616	0.90 (0.76–1.00)		0.72 (0.35–1.00)		NR	• The use of autofluorescence would result in more false-positive results when used in primary care settings
Kim [82]	OPMD, OSCC Specialist care	27	AF		2695	3104	0.82 (0.75–0.88)		0.62 (0.49–0.75)		8.20 (4.02–16.72)	• A significant difference in sensitivity was shown in the direct comparison of autofluorescence with clinical examination
Buenahora [73]	OPMD, OSCC NR	25	VELscope, EVINCE, GOCCLES and other methods		2149	5562	0.86 (0.77–0.91)		0.72 (0.61–0.81)		15.00 (7.00–33.00)	• Autofluorescence is simpler and faster compared to other techniques • Autofluorescence can favor the presumptive diagnosis op OPMD and OSCC
Moffa [78]	OPMD, OSCC NR	17	VELscope, Horus UOC100, Identafi, GOCCLES		2061	2076	0.81 (0.74–0.88)		0.52 (0.37–0.67)		5.44 (2.29–10.56)	• Autofluorescence seems to be an adequate adjunctive tool, increasing the execution of biopsies • Autofluorescence is adequate, even if the lesion is not suspected at COE • The use of autofluorescence improves the detection of early-stage tumors
Walsh [47]	OPMD, OSCC Specialist care	NR [83]	AF		NR	2140	0.88 (0.80–0.93)		0.61 (0.44–0.75)		NR	• The utility of this adjunct should be confined for use by expert clinicians to facilitate biopsy site selection in heterogeneous OPMDs or as a surveillance tool
Kim [74]	OPMD, OSCC NR	9	AF		659	933	0.86 (0.74 −0.92)		0.49 (0.29–0.69)		NR	• In evaluating obvious neoplastic lesions, there would be no difference between autofluorescence and clinical examination
Mendonca [49]	OPMD, OSCC NR	15	AF		NR	2033	0.75 (0.72–0.78)		0.50 (0.47–0.53)		3.65 (1.76–7.55)	• Inability to discriminate benign lesions from malignant lesions, due to higher false positive rates resulting in lower specificity
Flores dos Santos [84]	OPMD NR	10	VELscope		1308	NR	0.74 (0.68–0.80)		0.57 (0.53–0.60)		2.55 (0.85–7.68)	• Autofluorescence would provide a better visualization of tissue changes • Autofluorescence would indicate the most critical moment for a surgical intervention
*Optical imaging*												
Ansari [36]	OPMD, OSCC NR	4	NBI		548	NR	0.76 (0.65–0.84)		0.92 (0.82–0.96)		33.6	• NBI is a promising diagnostic and surveillance tool for suspicious lesions in the oral cavity
Kim [85]	OPMD, OSCC NR	2	WLE		NR	731	0.48 (0.61–0.99)		0.52 (0.41–0.63)		8.99 (0.001–1333.30)	• White light imaging (WLI) principally detects mucosal changes or mass lesions • WLI lacks the sensitivity required to screen for superficial cancers or precancerous lesions
		3	NBI		NR	638	0.87 (0.72–0.94)		0.95 (0.71–0.99)		57.76 (12.39–269.40)	• Using NBI in class I or II dysplastic lesions would be associated with high false-positive rates, unnecessary biopsies, or overtreatment • Using NBI in IPCL class III criteria lesions afforded good sensitivity and specificity
		3	NBI		NR	189	0.99 (0.61–1.00)		0.57 (0.31–0.79)		42.31 (3.08–582.16)	
		4	NBI		NR	594	0.60 (0.34–0.82)		0.99 (0.92–1.00)		53.80 (12.95–223.45)	
Kim [74]	OPMD, OSCC NR	2	NBI		224	224	0.90 (0.83–0.95)		0.95 (0.76–0.99)		NR	• NBI could differentiate superficial mucosal lesions not detected under standard white light imaging • NBI is a useful tool for early diagnosis of OPMD or cancerous lesions
Mendonca [49]	OPMD, OSCC NR	6	NBI		NR	593	0.31 (0.25–0.37)		0.90 (0.86–0.93)		4.12 (1.89–8.99)	• Meta-analysis shows that NBI could distinguish OSCC with higher diagnostic values
Kim [86]	OPMD, OSCC Specialist care	12	OCT		481	NR	0.91 (0.88–0.94)		0.91 (0.86–0.95)		86.92 (38.74–195.00)	• With an AUC of 0.951, OCT shows an excellent diagnostic accuracy
Zhang [87]	OPMD, OSCC NR	9	NBI		1179	NR	0.87 (0.67–0.96)		0.83 (0.56–0.95)		87.40 (8.62–886.50)	• NBI showed good diagnostic value in detecting oral lesions with dysplasia or cancer • Especially in lesions categorized with IPCL I diagnostic values were good
		9	NBI		1232	NR	0.58 (0.19–0.89)		0.95 (0.85–0.99)		28.38 (7.18–112.11)	
		6	NBI		946	NR	0.69 (0.47–0.85)		0.97 (0.88–0.99)		63.54 (20.17–200.19)	
		5	NBI		890	NR	0.97 (0.82- 0.99)		0.55 (0.23–0.83)		36.21 (5.38–243.74)	
*Imaging-based techniques*												
Moreira [88]	OSCC Specialist care	9	Diffuse-weighted MRI		NR	374	0.76 (0.67–0.84)		0.91 (0.87–0.94)		30.70 (12.70–74.30)	• The accuracy of MRI is good according to the DOR • MRI can be used for diagnosis and prevention of OSCC
		7	Dynamic contrast enhanced MRI		NR	337	0.84 (0.76–0.90)		0.90 (0.85–0.93)		48.10 (22.40–103.20)	
		13	traditional MRI		NR	1014	0.73 (0.66–0.78)		0.87 (0.84–0.89)		23.90 (13.20–43.30)	
*Spectroscopy-based techniques*												
Fuller [72]	OPMD, OSCC NR	NR [3]	DRS		NR	122	0.99 (0.94–1.00)		0.83 (0.63–0.95)		NR	• The overall accuracy was impressive • Due to a high prevalence of disease, the techniques are more sensitive than specific
Han [89]	OSCC NR	2	RS		99	NR	0.85 (0.83–0.87)		0.70 (0.67–0.73)		22,178.00	• Diagnostic performance is reliable to differentiate OSCC from normal oral samples
Mendonca [49]	OPMD NR	3	DRS		NR	240	0.79 (0.67–0.89)		0.86 (0.80–0.91)		40.47 (0.80–2047.56)	• Comparatively higher accuracy in diagnosis of OSCC was obtained • Higher pooled specificity values were shown in diagnosis of dysplasia
	OSCC NR	6	DRS		NR	411	0.93 (0.88–0.97)		0.90 (0.85–0.93)		NR	
Shrivastava [90]	OPMD, OSCC NR	8	RS		NR	NR	1.00 (0.72–1.00)		0.93 (0.80–0.98)		NR	• Consistent evidence was provided favoring the use of Raman Spectroscopy and FTIR in the early and accurate diagnosis of malignant and potentially malignant conditions of the oral cavity
	OPMD, OSCC NR	4	FTIR		NR	NR	0.98 (0.68–1.00)		0.96 (0.85–0.99)		NR	
*Cytology—brush techniques*												
Fuller [72]	OPMD, OSCC NR	NR [11]	OralCDx, brush, spatula		NR	1147	0.90 (0.83–0.95)		0.90 (0.83–0.95)		NR	• A statistically significant improvement in sensitivity and specificity was demonstrated of cytology over COE
Macey [76]	OPMD, OSCC Specialist care	12	OralCDx, Cytobrush, curette		1507	1575	0.91 (0.81–0.96)		0.91 (0.81–0.95)		NR	• Current cytology test outcomes cannot discriminate between epithelial dysplasia or OSCC • Tissue biopsy is still necessary for a definitive diagnosis
Ye [41]	OPMD, OSCC NR	8	OralCDx		1598	NR	0.86 (0.81–0.90)		0.81 (0.78–0.85)		20.36 (2.72–152.67)	• Brush biopsy has significant potential for diagnosis
Lingen [42]	OPMD, OSCC Specialist care	15	OralCDx, OralCyte, ClearPrep OC		NR	2148	0.92 (0.86–0.98)		0.94 (0.88–0.99)		NR	• Cytologic testing seems to be the most accurate adjunct compared to VS, Chemiluminescence and autofluorescence • Quality of evidence is low
Walsh [47]	OPMD, OSCC Specialist care	NR [3]	Oral brush		NR	417	0.93 (0.87–0.96)		0.92 (0.81–0.97)		NR	• At present it is not recommended as a replacement for histological assessment • Cytology would appear to offer the most potential compared to vital staining and light-based tests
		NR [91]	Oral brush		NR	1950	0.91 (0.81–0.96)		0.94 (0.87–0.97)		NR	
Kim [74]	OPMD, OSCC NR	3	Oral brush		171	179	0.72 (0.45–0.89)		0.86 (0.73–0.94)		NR	• Diagnostic accuracy tends to be higher than clinical examination • No significant difference between the oral brush and COE
*Computer-based techniques*												
Luo [81]	OPMD, OSCC Specialist care	8	AI + AF		NR	1068	0.92 (0.88–0.94)		0.95 (0.92–0.97)		194.11 (94.27–399.71)	• Promising use in primary care • Proper algorithms could be combined with autofluorescence to enhance its specificity
Elmakaty [58]	OSCC NR	5	AI + AF		NR	683	0.93 (0.86–0.97)		0.97 (0.92–0.99)		378.00 (126.00–1136.00)	• AI-assisted autofluorescence had the highest specificity compared to spectroscopy and photography
	OSCC NR	5	AI + RS		NR	1314	0.98 (0.96–0.99)		0.93 (0.88–0.96)		547.00 (262.00–1140.00)	• AI-assisted Raman spectroscopy had the best sensitivity compared to Autofluorescence and photography
	OSCC NR	7	AI + photography		NR	2525	0.90 (0.79–0.96)		0.93 (0.84–0.97)		130.00 (37.90–443.00)	• The use of oral images in diagnosing OSCC achieved good performance with a sensitivity of 90%
Kim [51]	OPMD, OSCC NR	4	AI + AF		NR	NR	0.86 (0.80–0.90)		0.84 (0.76–0.89)		27.63 (17.23–44.32)	• AI diagnosis sensitivity using autofluorescence images was confirmed to be 85% in all precancerous lesions • AF imaging is of sufficient value as an adjunct diagnostic tool
	OPMD, OSCC NR	3	AI + OCT		NR	NR	0.94 (0.80–0.99)		0.97 (0.87–0.99)		324.33 (10.25–10,261.60)	• AI diagnosis using OCT images is considered of sufficient value as a screening method for oral lesions
	OPMD, OSCC NR	5	AI + photography		NR	NR	0.91 (0.87–0.94)		0.88 (0.83–0.91)		66.81 (38.02–117.40)	• AI diagnosis from photographic images was confirmed to have a diagnostic sensitivity of over 91% for precancerous and cancerous lesions
Kim [86]	OPMD, OSCC Specialist care	5	AI + OCT		NR	NR	0.94 (0.87–0.98)		0.95 (0.87–0.98)		293.88 (37.17–2323.73)	• OCT-based screening for OSCC showed superior diagnostic accuracy compared to screening for OPMDs

*OPMD *oral potentially malignant disorders, *OSCC * oral squamous cell carcinoma, *OSF* oral submucous fibrosis, *OLP* oral lichen planus, *OL *oral leukoplakia, *95% CI *95% confidence interval, *NR* not reported, *COE *conventional oral examination, *CHEM* (chemiluminescence, *AF *autofluorescence, *TB *toluidine blue staining, *LI *Lugol's Iodine staining, *RB *Rose Bengal staining, *LIAF *laser induced autofluorescence, *OCT *optical coherence tomography, *RS *Raman spectroscopy, *DRS *diffuse reflectance spectroscopy, *FTIR *Fourier-transform infrared, *NBI *narrow-band imaging, *WLE *white light endoscopy, *AI *artificial intelligence, *NMA* Network Meta-analysis, *IPCL * intraepithelial papillary capillary loops

### Bivariate analysis

**Fig. 2:**
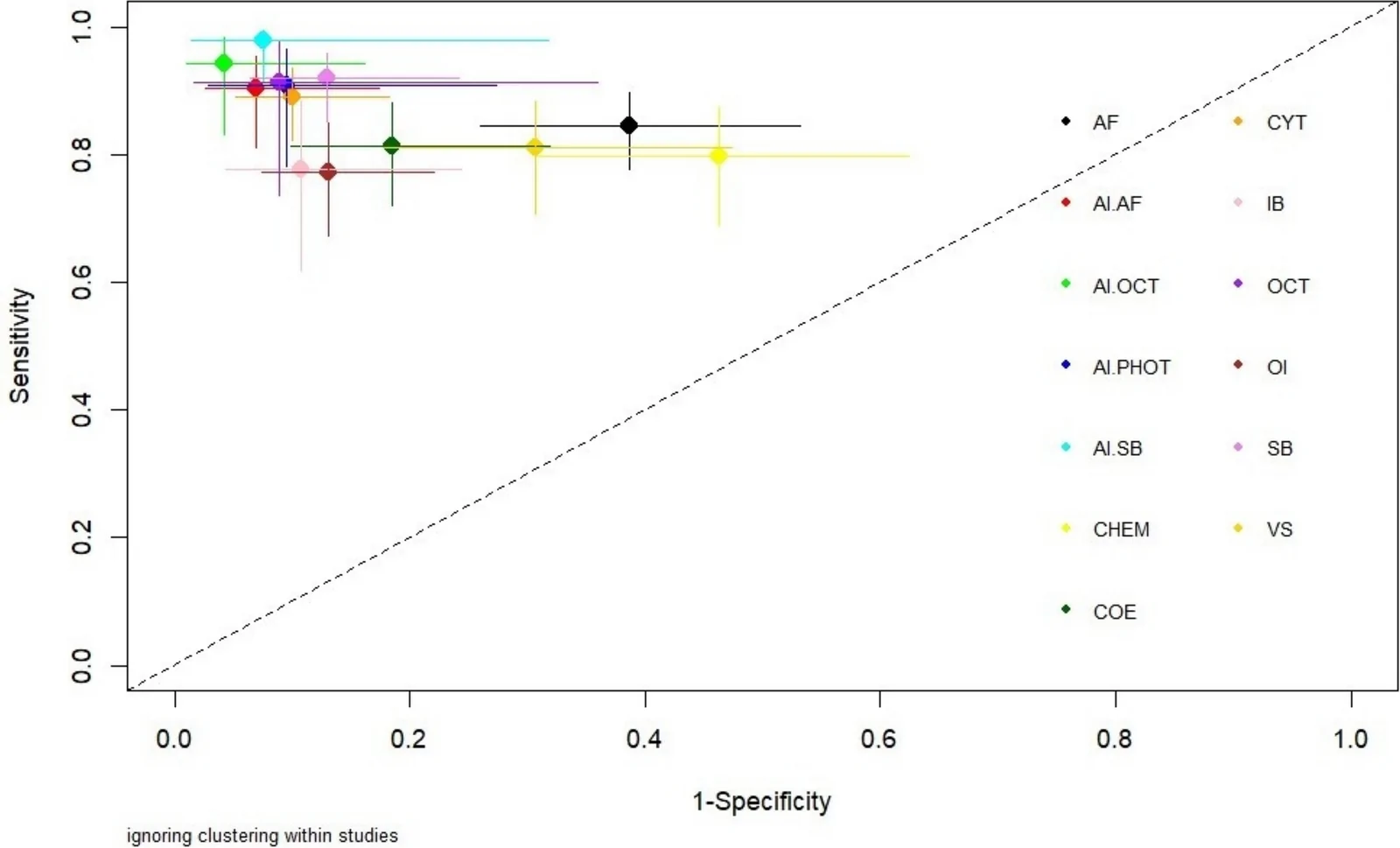
Mean sensitivity and specificity: jointly estimated bivariate model. *AF * autofluorescence, *AI.AF *AI + autofluorescence, *AI.OCT* AI + Optical Coherence Tomography, *AI.PHOT* AI + clinical photography, *AI.SB *AI + Spectroscopy, *CHEM * chemiluminescence, *COE * Conventional Oral Examination, *CYT * cytology- brush based, *IB * Imaging-based techniques, *OCT * Optical Coherence Tomography, *OI * Optical imaging, *SB * spectroscopy-based techniques, *VS * vital staining

Sensitivity, specificity, and DOR of each technique were analyzed through a bivariate analysis, based on a REM. Table 4 shows the pooled accuracy estimates per technique, including the number of included systematic reviews (k) and total number of primary studies (N) contributing to each analysis. Visual comparisons of sensitivity and specificity are presented in Fig. 2. **Spectroscopy** showed the highest pooled sensitivity without AI (0.92) with a specificity 0.87 and DOR of 78.7. When combined with AI, these values improved to 0.98, 0.93, and 548.3. **OCT** showed a relatively high sensitivity and specificity of 0.91, which also improved when combined with AI (0.94 and 0.96). With a comparable specificity of 0.90 and a sensitivity of 0.89, **cytology-based techniques** show a high DOR of 74.1, which is higher compared to most other evaluated techniques. In contrast, imaging-based techniques exhibited the lowest sensitivity (0.78).

**Table 4:** Bivariate analysis of sensitivity and specificity of the non-invasive diagnostic techniques

Technique	k(N)	Sens (95%CI)	Spec (95%CI)	DOR
COE	7(79)	0.81 (0.72–0.88)	0.82 (0.68–0.90)	19.3 (8.2–45.7)
Vital staining	6(100)	0.81 (0.71–0.88)	0.69 (0.53–0.82)	9.7 (4.0–23.1)
Chemiluminescence	8(92)	0.80 (0.69–0.87)	0.54 (0.38–0.69)	4.5 (1.9–10.5)
Autofluorescence	10(147)	0.85 (0.78–0.90)	0.61 (0.47 −0.74)	8.7 (4.3–17.7)
OCT	1(12)	0.91 (0.74–0.98)	0.91 (0.64–0.98)	108.5 (13.5–873.1)
Optical imaging	5(53)	0.77 (0.67–0.85)	0.87 (0.78–0.93)	22.5 (10.3–873.1)
Spectroscopy	4(26)	0.92 (0.85–0.90)	0.87 (0.76–0.94)	78.7 (29.1–49.1)
Cytology-based	6(69)	0.89 (0.82–0.94)	0.90 (0.82–0.95)	74.1 (31.3–175.6)
Imaging-based	4(42)	0.78 (0.62–0.88)	0.89 (0.76–0.96)	29.1 (8.9–95.3)

*k* number of systematic reviews included, *N* total number of primary studies included, *Sens* sensitivity, *Spec* specificity, *DOR* diagnostic odds ratio, *COE* conventional oral examination, *OCT* optical coherence tomography

**Fig. 3:**
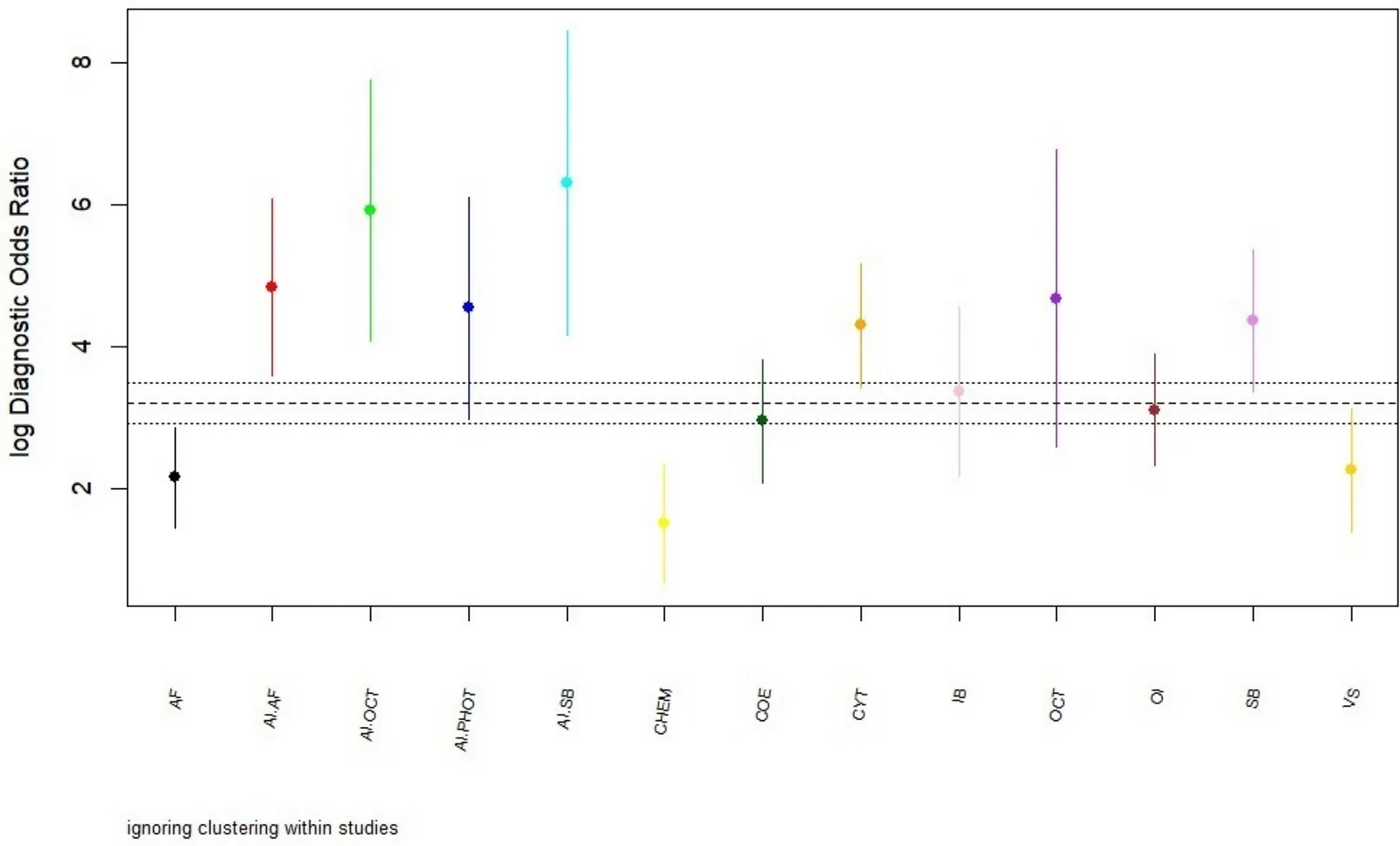
Mean logDOR: jointly estimated bivariate model. *AF * autofluorescence, *AI.AF * AI + autofluorescence, *AI.OCT * AI + Optical Coherence Tomography, *AI.PHOT * AI + clinical photography, *AI.SB * AI + Spectroscopy, *CHEM * chemiluminescence, *COE * Conventional Oral Examination, *CYT *cytology,brush based, *IB *Imaging-based techniques, *OCT *Optical Coherence Tomography, *OI * Optical imaging, *SB * spectroscopy-based techniques, *VS * vital staining

All techniques showed a DOR greater than one, with a mean DOR of 24.7 (Fig. 3). OCT showed the highest discriminative power (DOR = 108.5), whereas chemiluminescence showed the lowest (DOR = 4.5). Incorporating AI consistently improved the techniques, leading to higher sensitivity, specificity, and DOR values (Table 5). The bivariate analysis showed substantial heterogeneity in the logDOR estimates (Q = 68.44, df = 12, *p* < 0.001) with an I^2^ of 82.5%. To address the observed high heterogeneity, an attempt for subgroup analyses was done, however, as separating techniques and diseases resulted in subgroups containing only one or two studies, subgroup analyses were not feasible. The pairwise comparison (supplementary file 6) showed that of the tests without the use of AI, chemiluminescence showed the weakest diagnostic accuracy compared to other tests. Techniques with the use of AI significantly showed a higher diagnostic accuracy compared to the other tests.

**Table 5:** Bivariate analysis of sensitivity and specificity of the non-invasive diagnostic techniques combined with AI

Technique	k(N)	Sens (95%CI)	Spec (95%CI)	DOR
Autofluorescence	3(17)	0.90 (0.81–0.95)	0.93 (0.83–0.97)	126.2 (36.6–434.8)
Clinical photography	2(12)	0.91 (0.78–0.97)	0.91 (0.73–0.97)	94.9 (19.9–451.3)
OCT	2(8)	0.94 (0.83–0.98)	0.96 (0.84–0.99)	372.5 (59.7–2325.1)
Spectroscopy	1(1)	0.98 (0.92–0.99)	0.93 (0.68–0.99)	548.3 (64.9–4633.0)

*k* number of systematic reviews included, *N* total number of primary studies included, *Sens* sensitivity, *Spec* specificity, *DOR* diagnostic odds ratio, *AI* Artificial intelligence, *OCT* optical coherence tomography

### Multivariate meta regression analysis

A multivariate regression analysis was conducted, where test type was used as covariate (supplementary file 6). Sensitivity and specificity were modelled on logit scale, based on a bivariate random effects model, taking between-study variation and a correlation between the parameters into account (r = 0.99). Results of the multivariate meta regression analysis mainly showed comparable sensitivity and specificity values for all techniques as seen in the bivariate analysis. A slight change was observed in sensitivity and specificity values for AI-assisted spectroscopy and OCT, which showed the highest sensitivity (0.94, SD = 0.62 and 0.95, SD = 0.50, respectively) and high specificity (0.90, SD = 0.58 and 0.93, SD = 0.44, respectively). Notably, spectroscopy without the adjunctive use of AI showed an equal sensitivity compared to AI-assisted spectroscopy (0.95, SD = 0.50). Vital staining, chemiluminescence, autofluorescence, COE and imaging-based methods show relatively low values. Despite adding test type as covariate, residual heterogeneity was found (Q = 18.67, df = 12, *p* = 0.096, I^2^ = 35.8%).

### Publication bias

**Fig. 4:**
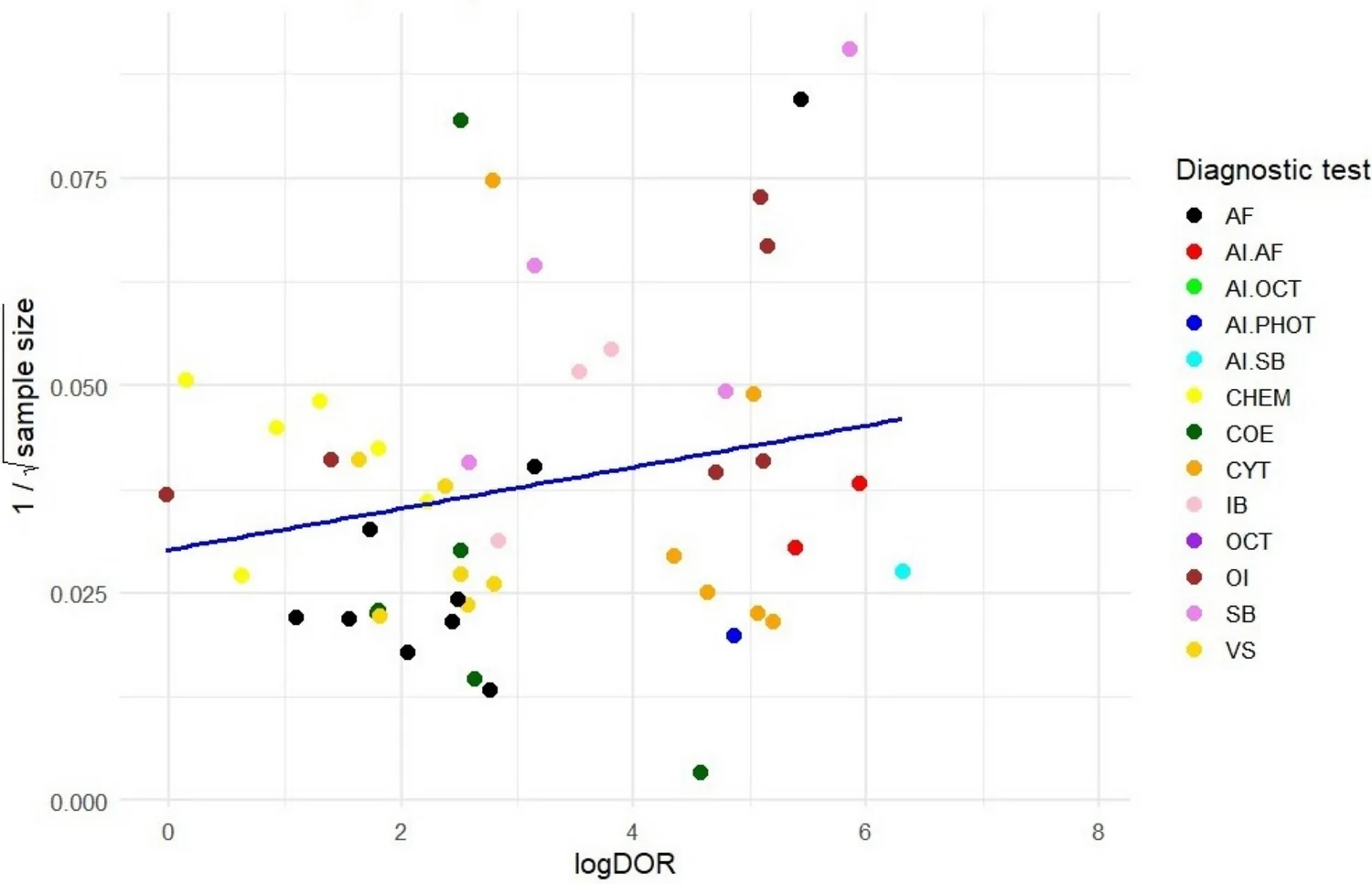
Deeks’ funnel plot, weighted model (*p* = 0.0198) *AF * autofluorescence, *AI *Artificial Intelligence. *AI.AF * AI + autofluorescence, *AI.OCT * AI + Optical Coherence Tomography, *AI.PHOT * AI + clinical photography, *AI.SB * AI + Spectroscopy, *CHEM * chemiluminescence, *COE * Conventional Oral Examination, *CYT * cytology- brush based; *IB * Imaging-based techniques, *OCT * Optical Coherence Tomography, *OI * Optical imaging, *SB * spectroscopy-based techniques, *VS * vital staining

Publication bias is visualized in Deeks’ funnel plot in Fig. 4. Visual inspection suggests a high asymmetry and Deek’s regression analysis showed a significant indication for small-review effects or meta-level publication bias (β = −62.21, SE = 8.95, p < 0.05) and indicated an association between study size and diagnostic accuracy. The CCA index was 1.79% for all included SRs, which can be interpreted as a slight overlap of the primary studies between the SRs according to Pieper et al. [92]. The CCA index for SRs that were included for quantitative analysis was estimated at 4.69%.

## Discussion

Our umbrella review aimed to evaluate clinically tested, non-invasive techniques for early detection and monitoring of OSCC and OPMDs on their efficacy. This study indicates that most evaluated non-invasive techniques are suggested to be best suited for specialist clinics, as their diagnostic accuracy often depends on the experience of the clinician. Among nine techniques that are available in practice for detecting OSCC and OPMDs, OCT, spectroscopy and cytology techniques with oral brushes, demonstrated the highest performance, specifically when combined with AI, diagnostic accuracies are even more improved. Besides the low sensitivity, optical imaging (NBI, WLE) and imaging-based methods (MRI) exhibited a high specificity. This suggests that these non-invasive techniques may have greater applicability in screening settings than in diagnostic settings.

Other studies that have compared adjunctive non-invasive techniques overlook recent advancements such as the use of cytology or biomarker systems in for example salivary tests, as well as the innovations in AI and deep learning. An umbrella review performed by Lau et al. in 2023 included 27 studies that were analyzed qualitatively [16]. They evaluated the use of these tools specifically in primary and secondary care, to determine their use as either an adjunctive tool or as a replacement of the gold standard. Another umbrella review conducted in 2023 by Shahnawaz Khijmatgar et al., evaluated the diagnostic value of specific different biomarkers for the detection of OSCC based on four included meta-analyses [93]. Our review expands on these two reviews, by including more studies on additional non-invasive techniques such as cytology and biomarker approaches based on salivary tests, as well as the inclusion of deep learning and AI models. In addition, this review provides a quantitative synthesis, offering a more comprehensive overview of diagnostic effectiveness. Quantitative analysis of molecular biomarkers could not be performed due to substantial inter-study heterogeneity. Diagnostic accuracy of molecular biomarkers varies, depending on their degree of association with the disease, the detection method used (e.g., DNA-ICM, DNA or RNA sequencing, PCR or ELISA), the sample type (e.g., saliva or oral brush), and whether a combination was used instead of individual biomarkers. The qualitative summary table completes this study.

This review highlights that spectroscopy and OCT, especially when combined with AI, demonstrate the highest sensitivity with narrow confidence intervals. Even without AI, these techniques outperform others like optical imaging and oral brush cytology. Although both the bivariate and Bayesian multivariate regression analyses account for the weight of included studies, it is noteworthy that the accuracy estimates of these two highest-scoring diagnostic techniques are based on the fewest included systematic reviews and primary studies, which warrants cautious interpretation of their accuracy estimates. Bivariate analysis revealed a higher sensitivity for the oral brush compared to optical imaging, and greater variability in sensitivity estimates for COE and vital staining. However, these findings should be interpreted cautiously, as the bivariate model is less robust, because it may underestimate uncertainty due to clustering [26].

The bivariate analysis and the Bayesian multivariate meta-regression yielded differing results, primarily due to how each method manages study-level clustering. While the bivariate approach treats all observations independently, the Bayesian model accounts for within-study correlations, offering more nuanced estimates. OCT and spectroscopy in combination with AI showed high sensitivity values and high specificity values. This implies a high detection rate and a low risk of false-positive results. By incorporating study-level clustering, the Bayesian model applies a shrinkage effect, pulling extreme values toward the overall mean. This results in more stable and robust parameter estimates, particularly in the presence of heterogeneous data.

Although AI- and telehealth-based screening methods were excluded from the main review to focus on clinically tested techniques, several included studies did incorporate AI components. To further explore this, an additional study was conducted using studies excluded from the main review [94]. This study demonstrated the potential of AI models to enhance early detection of OSCC and OPMDs, where non-invasive techniques combined with AI often achieved high diagnostic accuracy [51, 58, 94]. This is in line with our findings, where diagnostic techniques combined with AI showed a higher diagnostic accuracy. Particularly promising are approaches that integrate AI or machine learning with imaging modalities such as clinical photographs, which may offer an accessible solution for early detection, especially in low-resource countries where access to specialized care is challenging [53].

Sensitivity and specificity are standard measures of diagnostic accuracy, as they quantify the proportions of true positives and true negatives, respectively. In clinical practice, however, predictive values are often more relevant because they indicate the probability of disease given a specific test result. Unlike sensitivity and specificity, predictive values are strongly influenced by disease prevalence and pre-test probability, whereas sensitivity and specificity are generally more stable across populations [95]. Sensitivity and specificity are typically inversely related. In our review a positive correlation was observed in the HSROC analysis violating model assumptions and necessitating the use of a bivariate model [23]. Sparse data in meta-analyses can hinder convergence and produce unreliable estimates in HSROC models, and while they account for between-study variability, substantial heterogeneity may still compromise model fit [96, 97]. Substantial heterogeneity in our review supported the choice of a bivariate approach for summary estimation [96]. Variability in diagnostic positivity thresholds across studies likely contributed to the observed correlation between sensitivity and specificity.

SRs provide the highest level of evidence. To summarize and synthesize evidence in an umbrella review, consistent or contradictory evidence can be highlighted and it can be explored if similar results and conclusions can be found [20]. However, the validity of an umbrella review depends on the methodological quality of both the included SRs and the underlying primary studies. Despite the critically conducted inclusion of the SRs and meta-analyses with the Joanna Briggs Institutes quality assessment [20], the reliability seems to be questionable, since an unexpected positive correlation between sensitivity and specificity was found. A possible reason for this could be due to biases in primary studies, such as differences in thresholds or differences in test quality.

Another important factor causing possible unreliability in the accuracy values in this current meta-analysis, is that OPMDs encompass a diverse group of lesions, each with distinct clinical and histopathological characteristics. The diagnosis of OPMD is made clinically but can be supported histopathologically. In histopathological diagnosis, particular attention is paid to the presence of oral epithelial dysplasia, which serves as the most important indicator of malignant transformation (MT). However, the risk of MT among OPMDs is highly variable, ranging from 6.6% to 36.4%, which underlines the heterogeneity of these lesions [98]. Among OPMDs, OL is the most prevalent and has an estimated annual MT rate of 3–5% [83, 91]. Clinico-histopathological risk factors associated with progression include female gender, lesion size, homogeneity and the presence of oral epithelial dysplasia [10]. In contrast, oral erythroplakia exhibits a significantly higher MT rate, estimated between 20 and 68% annually, with severe epithelial dysplasia serving as a critical marker for malignant progression [99]. The malignant potential of OLP remains questionable, due to diagnostic challenges. Some dysplastic lesions present with a band-like lymphocytic infiltrate, mimicking OLP, which complicates the assessment of true MT risk [100]. The heterogeneity in clinical presentation and histopathological characteristics among OPMDs likely contributes to variability in diagnostic accuracy. Nevertheless, many studies evaluating the diagnostic performance of non-invasive tests for OPMDs and OSCC tend to aggregate these conditions without stratification by lesion type. This methodological limitation may introduce misclassification or dilution bias, thereby obscuring lesion-specific diagnostic performance, reducing the reliability of reported accuracy estimates, and potentially leading to an underestimation of true diagnostic differences between lesion types [101]. Consequently, no firm conclusions can currently be drawn regarding the diagnostic accuracy of these techniques for the detection of OSCC or specific OPMD subtypes, nor regarding their ability to predict malignant transformation into OSCC.

As emphasized by Odell et al. [12], dysplasia grading should support clinical decision-making by guiding patient management. Nevertheless, the dysplasia grades, ranging from mild to severe dysplasia (sometimes indicated as carcinoma in situ), and even architectural dysplasia, are not defined diagnostic categories. Studies have demonstrated considerable inter- and intra-observer variability in dysplasia grading, highlighting the subjectivity and complexity of using dysplasia as a histopathological endpoint. In 2022, architectural dysplasia was introduced as a distinct subtype of oral epithelial dysplasia. Architectural dysplasia, characterized by structural abnormalities in epithelial organization, has emerged as a strong predictor for malignant progression in OL. This recent understanding underlines the need for refined histopathological criteria and their integration into diagnostic frameworks. Incorporating such nuanced classifications may enhance the accuracy of diagnostic tests and improve risk stratification in clinical practice. These are crucial factors that need to be taken into account for future studies. Although a considerable degree of heterogeneity is common in meta-analysis of diagnostic accuracy studies, the inconsistency in classifications of the lesions likely contributed to the observed high heterogeneity of this current review [97, 102].

To address the observed high heterogeneity, subgroup analyses were attempted by separating OSCC and OPMDs. However, separating techniques and diseases led to subgroups of one or two studies to include in the meta-analysis. These subgroup datasets were too limited to support a statistically valid meta-analysis. Although this limitation prevents definitive conclusions, it is plausible that the lack of clear classification and lesion-specific evaluations contribute significantly to the heterogeneity observed across studies. This highlights the need for future research to distinguish between OSCC and individual OPMD types in both study design and analysis, to improve the reliability and interpretability of diagnostic accuracy outcomes. Importantly, consistent diagnostic criteria are also crucial for longitudinal studies investigating early detection of OPMD recurrence and malignant transformation [3].

In addition to quantitative measures, the clinical implementation and limitations of each technique, as well as availability, costs, and training requirements are relevant in this context. A study investigating barriers to the adoption of novel diagnostic tests in clinical practice reported that limited clinical applicability negatively affects both test accuracy and the likelihood of implementation in routine care [103]. OCT demonstrated the highest diagnostic accuracy among the evaluated techniques, but several studies emphasize that the clinical applicability of this technique is hampered due to the limited penetration depth resulting in underestimation of the lesion. Furthermore, accuracy values are also influenced by the level of experience of the operator [104, 105]. Among the spectroscopic techniques, RS is the most extensively studied, but this is mainly limited to small-scale in vivo pilot studies. A technical limitation of this technique includes a weak scattering signal which requires longer acquisition times to compensate. Portable devices have been developed, but still require validation in large clinical trials to demonstrate clinical implementation and cost-effectiveness [106].

Another possible limitation of an umbrella review, and therefore this study, is the risk of overlap in primary studies between the included SRs. Overlap in meta-reviews is a methodological issue that can lead to false assumptions about the evidence presented [107]. The calculated CCA index of all included reviews was 1.79% and for the included reviews for quantitative analysis. The CCA index was 4.69%, which suggests that some of the reviews shared overlapping primary studies, but at an acceptable level. Also, the slope of the regression line (−62.21) reflected significant association between study size and diagnostic accuracy [30, 31]. Therefore, the observed pattern in the funnel plot may not be reflected by dependency between reviews, but rather by small review effects or publication bias.

To summarize, the main limitations of this study include substantial heterogeneity among the included reviews; the frequent pooling of OSCC and OPMDs without lesion-specific stratification; inconsistencies in dysplasia grading across studies; and, for some diagnostic techniques, a limited number of available systematic reviews, which may reduce the reliability of the reported accuracy estimates.

## Conclusions

In this umbrella review nine non-invasive techniques used in clinical practice for the detection of OSCC and OPMDs have been evaluated on effectiveness and accuracy. Oral brush techniques showed promising diagnostic performance for detecting severe dysplasia and OSCC, although the available evidence remains insufficient to draw definitive conclusions regarding their reliability. OCT and spectroscopy demonstrated the highest diagnostic performance, with further improvements in accuracy reported when combined with AI. These estimates should be interpreted cautiously, as they are based on a limited number of systematic reviews.

It is important to emphasize that these findings cannot be directly extrapolated to the detection of OSCC or OPMDs themselves, as evidence regarding the diagnostic performance of these techniques in these specific clinical contexts remains limited. Furthermore, the results suggest that most evaluated non-invasive techniques are best suited for use in specialist clinical settings, where diagnostic accuracy is likely influenced by operator experience, underscoring the importance of clinical applicability when interpreting test performance.

No clinical guidelines are currently available for management of OPMDs, and practical guidelines for clinical decision making in the field of oral cancer and oral potentially malignant disorders will be helpful. More high-quality primary studies are thus needed to evaluate the diagnostic accuracy of different diagnostic modalities, including AI–assisted approaches and molecular biomarker–based tests, specifically stratified by lesion type. In addition, such studies should ideally be conducted in specialist clinical settings to ensure optimal and clinically relevant performance assessment.

Accordingly, no definitive conclusions can currently be drawn regarding the diagnostic accuracy of these techniques for the detection of OSCC or different types of OPMDs, nor regarding their ability to predict malignant transformation into OSCC.

## Supplementary Information

Below is the link to the electronic supplementary material.

Supplementary file1 (DOCX 506 KB)

## Data Availability

The data that support the findings of this study are openly available in Figshare at https://doi.org/10.6084/m9.figshare.33327276.

## Abbreviations

OPMD: Oral potentially malignant disorder
OSCC: Oral squamous cell carcinoma
OL: Oral leukoplakia
PVL: Proliferative verrucous leukoplakia
OLP: Oral lichen planus
OSF: Oral Submucous Fibrosis
COE: Conventional oral examination
AI: Artificial intelligence
PRISMA: Preferred Reporting Items for Systematic Reviews and Meta-Analyses
PICOS: Population, Intervention, Comparison, Outcomes and Study type
SR: Systematic review
JBI: Joanna Briggs Institute
DOR: Diagnostic odds ratio
HSROC: Hierarchical Summary Receiver Operating Characteristic
Sens: Sensitivity
Spec: Specificity
MCMC: Markov chain Monte Carlo
CCA: Corrected Covered Area
TB: Toluidine blue
CHEM: Chemiluminescence
HRME: High resolution micro endoscopy
NBI: Narrow band imaging
AF: Autofluorescence
WLE: White light endoscopy
OCT: Optical coherence tomography
CLE: Confocal laser endomicroscopy
MRI: Magnetic resonance imaging
ESS: Elastic scattering spectroscopy
RS: Raman spectroscopy
DNA-ICM: DNA-image cytometry
PCR: Polymerase chain reaction
ELISA: Enzyme-linked immunosorbent assay
MT: Malignant transformation
