# Supplementary material for: Non-invasive detection and monitoring of oral squamous cell carcinoma and oral potentially malignant disorders: an umbrella review on diagnostic accuracy
Source: Clin Oral Investig. 2026 Aug 28;30(9):421. doi: 10.1007/s00784-026-07109-x (PMC13524989; doi:10.1007/s00784-026-07109-x)
Supplement: Supplementary file 1 [file 784_2026_7109_MOESM1_ESM.docx]

**Supplementary file 1 Search strategy**

A structured search strategy was performed using search terms and MeSH terms in PubMed, PsycINFO, and MEDLINE.

Search terms

- Patient: oral leukoplakia/oral precancer
- “Leukoplakia or oral mucosa”, “leukoplakia of mouth”, “leukoplakia of the oral”, “leukoplakia oral”, “Leukoplakia,Oral”, “Leukoplakias, Oral”, “Dysplastic oral leukoplakia”, “oral dysplasia”, “oral malignant lesion”
- Intervention: non-invasive screening tests
- “diag tests”, “test, diagnostic”, “oral screening devices”
- Outcome: detection
- “Detected”, “Detection”, “diagnostic procedure”

MeSH terms

- “Leukoplakia, Oral”
- “squamous cell carcinoma of head and neck” [MeSH Terms]
- “oral squamous cell carcinoma”
- “early detection of cancer”[MeSH]

PubMed search strategy

PsychINFO search strategy

1.	Leukoplakia, oral.ab,kf,ti

2.	(diagnosis or diagn* or detect*).ab,kf,ti

3.	Detection,early.ab,kf,ti.

4.	2 or 3

5.	1 and 4

6.	5 and systematic review

Medline via OVID search strategy

1.	(leukplakia, oral or oral malignant* lesion or mouth neoplasms or carcinoma, squamous cell or leukoplakia* or oral dysplasia*).ab,kf,ti

2.	(detect* or test, diagnostic or diagn* or oral screening device).ab,kf,ti

3.	1 and 2

4.	3 and systematic review.pt

**Supplementary file 2 List of excluded studies**

Studies that were excluded during study selection and quality assessment are listed below with reason for exclusion.

| **Author (year)** | **Reason for exclusion** |
| --- | --- |
| Lokesh Kumar et al. (2023) | Incorrect outcome |
| de Almeida Barros Mourão et al. (2022) | Incorrect publication type |
| Khong et al. (2024) | Incorrect outcome |
| Li et al. (2024) | Incorrect outcome |
| Lau et al. (2024) | Incorrect publication type (umbrella review) |
| Khijmatgar et al. (2024) | Incorrect publication type (umbrella review) |
| Gupta et al. (2023) | Incorrect outcome |
| Uhrin et al. (2023) | Discrepant results in abstract and full-text, unclear methodology |
| Araújo et al. (2023) | Incorrect publication type (umbrella review) |
| Mills et al. (2022) | Incorrect publication type |
| Gaw et al. (2022) | Incorrect publication type |
| Chasma et al. (2022) | Incorrect publication type |
| Adam et al. (2022) | Incorrect publication type |
| Richards et al. (2015) | Incorrect publication type |
| Carreras-Torras et al. (2015) | Incorrect study design |
| Kujan et al. (2005) | Incorrect study design |
| Chen et al. (2024) | Incorrect outcome |
| Liew et al. (2023) | Incorrect publication type, Incorrect study design |
| Menini et al. (2021) | Incorrect study design, Incorrect outcome |
| Chiamulera et al. (2021) | Incorrect study design |
| Arroyo et al. (2021) | Incorrect outcome |
| Tian et al. (2015) | Incorrect inclusion |
| Rapado-González et al. (2020) | Incorrect population |
| Gualtero et al. (2016) | Incorrect outcome |
| Patil et al. (2019) | Incorrect outcome |
| Hema Shree et al. (2019) | Insufficient methodological quality |
| Chaitanya et al. (2019) | Insufficient methodological quality |
| Cicciù et al. (2019) | Insufficient methodological quality |
| Bustiuc et al. (2018) | Insufficient methodological quality |
| Rosenberg et al. (1989) | Insufficient methodological quality |
| Yeladandi et al. (2023) | Insufficient methodological quality |
| Nagi et al. (2016) | Insufficient methodological quality |
| Giovannacci et al. (2016) | Insufficient methodological quality |
| Awan et al. (2015) | Insufficient methodological quality |

**Supplementary file 3 JBI critical appraisal checklist for Systematic Reviews and Research Synthesis**

Studies were scored based on the JBI critical appraisal checklist. One point for every question answered with “Yes”. To ensure the inclusion of studies with at least moderate to high methodological quality, those scoring 3 or lower were excluded.

Criteria:

1. Is the review question clearly and explicitly stated?
2. Were the inclusion criteria appropriate for the review question?
3. Was the search strategy appropriate?
4. Were the sources and resources used to search for studies adequate?
5. Were the criteria for appraising studies appropriate?
6. Was critical appraisal conducted by two or more reviewers independently?
7. Were there methods to minimize errors in data extraction?
8. Were the methods used to combine studies appropriate?
9. Was the likelihood of publication bias assessed?
10. Were recommendations for policy and/or practice supported by the reported data?
11. Were the specific directives for new research appropriate?

| **Criteria** | **1** | **2** | **3** | **4** | **5** | **6** | **7** | **8** | **9** | **10** | **11** | **Score** |
| --- | --- | --- | --- | --- | --- | --- | --- | --- | --- | --- | --- | --- |
| Studies |  |  |  |  |  |  |  |  |  |  |  |  |
| Patton (2003) [38] | Yes | Yes | Yes | No | Yes | No | No | NA | No | Yes | Yes | **6** |
| Patton (2008) [37] | Yes | Yes | Yes | Yes | Yes | Yes | No | NA | No | Yes | Yes | **8** |
| Epstein (2012) [5] | No | Yes | No | Yes | Yes | Unclear | Yes | Yes | Yes | NA | Yes | **7** |
| Walsh (2013) [34] | Yes | Yes | Yes | Yes | Yes | Unclear | Yes | NA | NA | Yes | Yes | **8** |
| Rashid (2015) [40] | No | Yes | Yes | Yes | Yes | Unclear | Yes | Yes | Yes | Yes | Yes | **9** |
| Omar (2015) [39] | Yes | Yes | Yes | No | Yes | No | No | NA | No | Yes | NA | **5** |
| Fuller (2015) [72] | No | No | Yes | Yes | Unclear | NA | Yes | Yes | No | NA | Yes | **5** |
| Macey (2015) [76] | Yes | Yes | Yes | Yes | Yes | Yes | Yes | Yes | No | Yes | Yes | **10** |
| Awan (2015) | Yes | Yes | Yes | No | Unclear | NA | No | NA | No | NA | NA | **3** |
| Ye (2015) [41] | No | Yes | Yes | Yes | Yes | No | Yes | Yes | Yes | Yes | NA | **8** |
| Nagi (2016) | No | No | No | No | Unclear | NA | No | NA | No | Yes | Yes | **2** |
| Giovannacci (2016) | No | Unclear | No | No | Yes | No | No | No | No | NA | Yes | **2** |
| Luo (2016) [81] | No | Yes | Yes | Yes | Yes | Unclear | Yes | Yes | Yes | Yes | Yes | **9** |
| Moreira (2017) [88] | No | Yes | Yes | Yes | Yes | Unclear | Yes | Yes | No | Yes | Yes | **8** |
| Lingen (2017) [42] | No | Yes | Yes | Yes | Yes | Yes | Yes | Unclear | No | Yes | NA | **7** |
| Alsarraf (2018) [43] | No | Yes | Yes | Yes | No | Unclear | Unclear | NA | NA | NA | Yes | **4** |
| Ansari (2019) [36] | No | Yes | Yes | Yes | Yes | Yes | Unclear | Yes | No | Yes | Yes | **8** |
| Datta (2019) [54] | Yes | Yes | Yes | No | Yes | Unclear | Yes | NA | NA | NA | Yes | **6** |
| Hema Shree (2019) | No | No | No | Yes | Unclear | NA | No | No | No | NA | Yes | **2** |
| Rapado-González (2019) [62] | No | Yes | Yes | Yes | Yes | Yes | Yes | Yes | Yes | NA | Yes | **9** |
| Kim DH (2020) [82] | No | Yes | Yes | Yes | Yes | Unclear | Unclear | Unclear | No | NA | Yes | **5** |
| Tiwari (2020) [44] | No | Yes | Yes | Yes | Yes | Yes | Yes | NA | NA | Yes | Yes | **8** |
| AlAli (2020) [63] | Yes | Yes | Yes | No | Yes | Unclear | No | NA | NA | NA | Yes | **5** |
| Buenahora (2021) [73] | No | Yes | Yes | No | Yes | Unclear | Unclear | Yes | No | Yes | Yes | **6** |
| Mazur (2021) [45] | No | Yes | Yes | Yes | Yes | Unclear | Yes | Yes | Yes | Yes | Yes | **9** |
| Moffa (2021) [78] | No | Yes | Yes | Yes | Yes | Yes | No | Yes | Yes | Yes | Yes | **9** |
| Kim DH (2021a) [77] | No | Yes | Yes | Yes | Yes | Unclear | No | Yes | No | Yes | Yes | **7** |
| Saraniti (2021) [46] | No | Yes | Yes | No | Unclear | NA | No | NA | NA | Yes | Yes | **4** |
| Lima (2021) [56] | Yes | Yes | Yes | Yes | Yes | Yes | Yes | NA | NA | Yes | Yes | **9** |
| Kim DH (2021b) [85] | No | Yes | Yes | Yes | Yes | Unclear | Yes | Yes | No | NA | Yes | **7** |
| Alabi (2021) [55] | No | Yes | Yes | Yes | Yes | Unclear | Yes | NA | NA | Yes | Yes | **7** |
| Walsh (2021a) [47] | Yes | Yes | Yes | Yes | Yes | Yes | Yes | Yes | No | Yes | Yes | **10** |
| Sethi (2021) [57] | No | Yes | Yes | Yes | Yes | Unclear | No | Yes | No | NA | Yes | **6** |
| Walsh (2021b) [35] | Yes | Yes | Yes | Yes | Yes | Unclear | Yes | NA | NA | No | Yes | **7** |
| Kim DH (2022a) [79] | No | Yes | Yes | Yes | Yes | Unclear | No | Unclear | No | NA | Yes | **5** |
| Shaw (2022a) [80] | Yes | Yes | Yes | Yes | Yes | Unclear | Yes | Unclear | No | Yes | Yes | **8** |
| Kim JS (2022) [51] | No | Yes | Yes | No | Yes | Unclear | No | Yes | Yes | NA | Yes | **6** |
| Kim DH (2022b) [74] | No | Yes | Yes | Yes | Yes | Unclear | Yes | Yes | Yes | Yes | Yes | **9** |
| Mendonca (2022) [49] | No | Yes | Yes | Yes | Yes | Yes | Yes | Yes | Yes | Yes | Yes | **10** |
| Zhang (2022) [87] | No | Yes | Yes | Yes | Yes | Yes | Yes | Yes | Yes | Yes | Yes | **10** |
| Han (2022) [89] | No | Yes | Yes | Yes | Yes | Unclear | Yes | Yes | Yes | NA | Yes | **8** |
| Adeoye (2022) [65] | No | Yes | Yes | Yes | Yes | Yes | Yes | Yes | Yes | NA | Yes | **9** |
| Essat (2022) [48] | No | Yes | No | Yes | Yes | Unclear | No | Yes | No | Yes | Yes | **6** |
| Shaw (2022b) [69] | Yes | Yes | Yes | Yes | Yes | Unclear | Yes | Unclear | No | NA | Yes | **7** |
| Dos Santos (2022) [84] | Yes | Yes | Yes | Yes | Yes | Yes | Yes | Yes | No | Yes | Yes | **10** |
| Elmakaty (2022) [58] | No | Yes | Yes | Yes | Yes | Yes | Yes | Yes | Yes | Yes | Yes | **9** |
| Sulaiman (2022) [75] | No | Yes | Yes | Yes | Yes | Unclear | Yes | Unclear | No | Yes | NA | **6** |
| Shrivastava (2023) [90] | No | Yes | Yes | Yes | Yes | Yes | Yes | Yes | Yes | NA | NA | **8** |
| Kim DH (2023) [86] | No | Yes | Yes | No | Yes | Unclear | Unclear | Yes | Yes | NA | Yes | **6** |
| Yeladandi (2023) | No | Yes | Yes | No | Unclear | NA | No | NA | NA | Yes | NA | **3** |
| Ramani (2023) [50] | No | Yes | Yes | No | Yes | Yes | No | NA | NA | Yes | Yes | **6** |
| Beristain-Colorado (2024) [52] | Yes | Yes | Yes | Yes | Yes | Yes | Yes | NA | NA | Yes | Yes | **9** |
| Li (2024) [53] | No | Yes | Yes | No | Yes | Yes | Yes | Yes | No | Yes | Yes | **8** |
| Gurushanth (2024) [71] | Yes | Yes | Yes | Yes | Yes | Yes | Unclear | Unclear | Yes | Yes | Yes | **9** |
| Kang (2021) [66] | No | Yes | Yes | No | Yes | Yes | Yes | Yes | Yes | NA | Yes | **8** |
| Guerra (2015) [59] | No | Yes | Yes | Yes | Yes | Yes | Yes | Unclear | No | NA | Yes | **7** |
| Liu (2021) [67] | No | Yes | Yes | No | Yes | Yes | Yes | Yes | Yes | NA | Yes | **8** |
| Bustiuc (2018) | No | Unclear | Unclear | No | Unclear | NA | No | NA | NA | NA | NA | **0** |
| Cicciù (2019) | Yes | No | No | No | Unclear | NA | Unclear | Unclear | No | NA | NA | **1** |
| Chaitanya (2019) | No | Unclear | Yes | No | Unclear | NA | No | Unclear | No | NA | NA | **1** |
| Tian (2015) [60] | No | Yes | Yes | Yes | Yes | Yes | Yes | Yes | No | NA | Yes | **8** |
| Rapado-González (2021) [68] | No | Yes | Yes | Yes | Yes | Yes | Yes | Yes | Yes | NA | Yes | **9** |
| Maheswari (2018) [61] | No | Yes | Yes | Unclear | Yes | Unclear | Unclear | NA | NA | NA | Yes | **4** |
| Rosenberg (1989) | No | Unclear | Unclear | Unclear | Unclear | NA | Unclear | Unclear | No | NA | Yes | **1** |
| Assad (2020) [64] | No | Yes | Yes | Yes | Yes | Yes | No | NA | NA | NA | Yes | **6** |
| Gaba (2021) [70] | No | Yes | Unclear | No | Yes | Yes | No | No | No | Yes | Yes | **5** |

**Supplementary file 4 Overlap of primary studies among included SRs**

Visualization of overlap of primary studies between SRs with at least 2 studies that overlap. Each row of dots represents one SR. The blue bars show the number of primary studies that overlap with primary studies that are included in another SR.

**Supplementary file 5 – Overview of non-invasive diagnostic techniques**

| **Category** | **Sub-technique** | **Description** |
| --- | --- | --- |
| **Conventional Oral Examination** | Oral examination | Inspection of the oral cavity. Gold standard first-line. It depends heavily on clinician experience. |
|  | Self-examination | Self-inspection of the mouth. |
| **Vital staining** | Toluidine blue | Also known as tolonium chloride. Dye stains tissues with increased nucleic acid content. |
|  | Rose Bengal | Stains dead or degenerated cells, but not healthy epithelial cells. |
|  | Lugol's Iodine | Normal epithelium stains brown due to its high glycogen content, abnormal tissue remains unstained. |
| **Light-based techniques** | Chemiluminescence | Uses acetic acid rinse and chemiluminescent light to enhance contrast between normal and abnormal mucosa, as mucosal lesions have different absorbance and reflectance profiles. |
|  | Autofluorescence | Excites endogenous tissue fluorophores, where abnormal mucosa shows decreased autofluorescence. |
| **Imaging techniques** | Narrow Band Imaging | Uses blue and green light to reveal vascular changes in superficial, abnormal mucosal lesions. |
|  | Optical Coherence Tomography | High-resolution microstructural imaging provides cross-sectional images to map the mucosal epithelium using near infrared light. |
|  | Magnetic resonance imaging | High-quality images of soft tissue. |
|  | Hyperspectral imaging | Captures wide wavelengths to distinguish tissue types. |
|  | Confocal laser endomicroscopy | Provides high subcellular resolution, real-time microscopic images of mucosa in vivo with limited penetration depth. |
|  | White light endoscopy (broadband white light) | Direct visualization of mucosal surfaces with conventional endoscopy. |
|  | High-resolution micro endoscopy | Portable device for cellular-level imaging of mucosa. |
|  | Raman spectroscopy | Identifies biochemical composition of tissues with inelastic scattering of light to provide specific characteristics of the structure and shape of the cells, known as “fingerprint molecules”. |
|  | Fourier-transform infrared | Measures absorption wavelengths resulting from vibrating molecular bonds (depending on chemical profile of a specific tissue). |
|  | Diffuse reflectance spectroscopy | Evaluates scattered light patterns to assess tissue morphology and absorbent elements (for example oxygenation). |
|  | Elastic scattering spectroscopy | Analysis of scattering changes gives structural information such as subcellular structure alterations. |
| **Cytology** | Oral brush and conventional cytology | Harvest transepithelial cells using a brush for cytological analysis. |
|  | Liquid-based cytology | Immediate fixation of cells and different preparatory technique with clear background and more homogenous thin layer of cells compared to conventional technique. |
| **Molecular biomarkers** | DNA image cytometry | Can be applied on oral brush cytology. Measures DNA content and ploidy in cells to identify aneuploidy. |
|  | Salivary (epi)genomic, transcriptomic, proteomic, metabolomic profiling | Identifies altered expression of microRNAs/genes/proteins/metabolites associated with malignant transformation. |

**Supplementary file 6 Pairwise comparison of all tests compared to all others in logDOR.**

Significant pairwise differences in logDOR (α – 0.001) shown in red. AF: autofluorescence; AI: Artificial Intelligence; AI.AF: AI + autofluorescence, AI.OCT: AI + Optical Coherence Tomography; AI.PHOT: AI + clinical photography; AI.SB: AI + Spectroscopy; CHEM: chemiluminescence; COE: Conventional Oral Examination; CYT: cytology, brush based; IB: Imaging based techniques; OCT: Optical Coherence Tomography; OI: Optical imaging; SB: spectroscopy based techniques; VS: vital staining
